# Genetic Mapping of Specific Interactions between *Aedes aegypti* Mosquitoes and Dengue Viruses

**DOI:** 10.1371/journal.pgen.1003621

**Published:** 2013-08-01

**Authors:** Thanyalak Fansiri, Albin Fontaine, Laure Diancourt, Valérie Caro, Butsaya Thaisomboonsuk, Jason H. Richardson, Richard G. Jarman, Alongkot Ponlawat, Louis Lambrechts

**Affiliations:** 1 Institut Pasteur, Centre National de la Recherche Scientifique, Unité de Recherche Associée 3012, Paris, France; 2 Department of Entomology, Armed Forces Research Institute of Medical Sciences, Bangkok, Thailand; 3 Institut Pasteur, Genotyping of Pathogens and Public Health, Paris, France; 4 Department of Virology, Armed Forces Research Institute of Medical Sciences, Bangkok, Thailand; National Institutes of Health, NIAID, United States of America

## Abstract

Specific interactions between host genotypes and pathogen genotypes (G×G interactions) are commonly observed in invertebrate systems. Such specificity challenges our current understanding of invertebrate defenses against pathogens because it contrasts the limited discriminatory power of known invertebrate immune responses. Lack of a mechanistic explanation, however, has questioned the nature of host factors underlying G×G interactions. In this study, we aimed to determine whether G×G interactions observed between dengue viruses and their *Aedes aegypti* vectors in nature can be mapped to discrete loci in the mosquito genome and to document their genetic architecture. We developed an innovative genetic mapping strategy to survey G×G interactions using outbred mosquito families that were experimentally exposed to genetically distinct isolates of two dengue virus serotypes derived from human patients. Genetic loci associated with vector competence indices were detected in multiple regions of the mosquito genome. Importantly, correlation between genotype and phenotype was virus isolate-specific at several of these loci, indicating G×G interactions. The relatively high percentage of phenotypic variation explained by the markers associated with G×G interactions (ranging from 7.8% to 16.5%) is consistent with large-effect host genetic factors. Our data demonstrate that G×G interactions between dengue viruses and mosquito vectors can be assigned to physical regions of the mosquito genome, some of which have a large effect on the phenotype. This finding establishes the existence of tangible host genetic factors underlying specific interactions between invertebrates and their pathogens in a natural system. Fine mapping of the uncovered genetic loci will elucidate the molecular mechanisms of mosquito-virus specificity.

## Author Summary

The outcome of invertebrate host-pathogen interactions often depends on the specific pairing of host and pathogen genotypes. This genetic specificity challenges our current understanding of invertebrate resistance to pathogens because it contrasts the limited discriminatory power of known invertebrate defense mechanisms. However, genetic factors underlying this observed specificity have remained elusive, questioning their very existence. In this study, we developed an innovative strategy to localize factors in the genome of the mosquito *Aedes aegypti* that govern specific interactions with dengue viruses. We used large mosquito families derived from a natural population in Thailand that we experimentally challenged with virus isolates obtained from human patients living in the same area. We identified several regions of the mosquito genome that control specific interactions with dengue viruses and contribute significantly to the observed variation in vector competence. Our study establishes the existence of tangible host genetic factors underlying specific interactions between invertebrates and their pathogens in a natural system that is relevant to human health. This represents a critical step towards identification of mechanisms underlying the genetic specificity of insect-virus interactions.

## Introduction

Most organisms engage in ecological interactions with organisms of different species that have profound effects on their fitness. These interactions, which can be antagonistic (e.g., parasitism, competition) or mutualistic (e.g., cooperation), are major drivers of adaptive evolution and diversification. Understanding the evolution of traits mediating ecological interactions can be complicated by their genetic specificity, whereby fitness of a genotype depends on the genotype of the interacting species [1], [2]. Such genotype-by-genotype (G×G) interactions, sometimes referred to as intergenomic epistasis, occur in both antagonistic [3] and mutualistic [4] relationships. Importantly, G×G interactions imply that the genetic basis of interaction traits is a composite entity that involves distinct genomes. Therefore, dissecting the genetic architecture (i.e., the number, position, effect and interactions between genetic loci underlying the phenotype) of these traits requires accounting jointly for genetic variation in different species [5].

Among the most intriguing examples of G×G interactions are those involved in invertebrate host susceptibility to pathogens [6]. Indeed, specific interactions between host and pathogen genotypes have been documented in a wide variety of invertebrate systems [7]–[12]. This observation challenges the long-held view that invertebrate defense against pathogens relies on broad-spectrum recognition and effector mechanisms [13], [14]. Lack of a mechanistic explanation, however, has questioned the nature of host factors underlying the observed G×G interactions [15]. For instance, the effect of host genotype can be confounded with that of symbiotic microbiota [16], raising the possibility that G×G interactions may be environmentally driven. A critical question is whether G×G interactions observed at the phenotypic level truly result from the effect of discrete genetic factors within host and pathogen genomes. More generally, understanding the ecological and evolutionary dynamics of host-pathogen interactions requires a detailed knowledge of their genetic architecture [17]. In this study, we addressed this question in a natural insect-virus association that is relevant for human health.

*Aedes aegypti* mosquitoes are the main vectors of dengue viruses, which cause the most prevalent mosquito-borne viral disease of humans [18]. Successful virus transmission requires that following mosquito blood feeding on a viremic host, infection is initially established in the insect's midgut cells and then disseminates throughout the rest of the body. The mosquito becomes infectious when the virus reaches the salivary glands and is released into the saliva. Vector competence defines the intrinsic ability of a mosquito to become infected following ingestion of infectious blood and to subsequently transmit the virus [19]. It varies substantially between and within *Ae. aegypti* populations throughout their wide geographical range [20], [21]. The existence of genetic factors underlying the observed variation in mosquito susceptibility to dengue was initially demonstrated by artificial selection of resistant and susceptible inbred lines of *Ae. aegypti* [22]. This finding confirmed that, as for many other host-pathogen systems [17], *Ae. aegypti* susceptibility to dengue has a genetic basis. Subsequent studies based on laboratory crosses of resistant and susceptible mosquito lines mapped several quantitative trait loci (QTL) controlling *Ae. aegypti* susceptibility to dengue virus infection and dissemination [23]–[25]. These QTL mapping studies, however, ignored the influence of viral genetic factors by exposing mosquitoes to a single, reference virus strain. A meta-analysis on the genetic architecture of host susceptibility in plants and animals revealed that QTL are recovered in only 25% of the cases when the mapping involves a different pathogen strain [17]. Dengue viruses exist in nature as four antigenically distinct serotypes (DENV-1 through DENV-4), which, in turn, consist of considerable genetic diversity [26]. Recently, we reported that several indices of *Ae. aegypti* vector competence for dengue viruses are governed by G×G interactions [9], [27]. Thus, the efficiency of dengue virus transmission by *Ae. aegypti* depends on the specific pairing of mosquito and virus genotypes.

**Figure 1:**
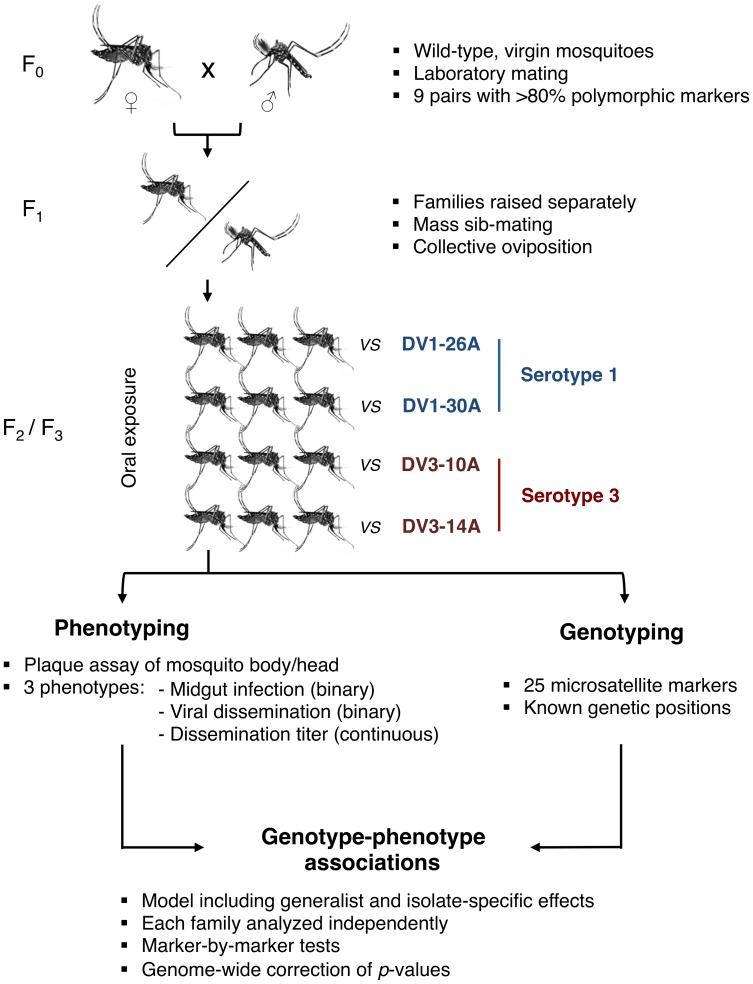
Schematic of the experimental strategy used to perform QTL mapping in an outbred *Ae. aegypti* population exposed to different dengue virus serotypes/isolates.

Here, we surveyed genetic factors within the *Ae. aegypti* genome that are associated with G×G interactions influencing vector competence for dengue viruses. We developed an innovative genetic mapping strategy (Fig. 1) based on wild-type *Ae. aegypti* families that were experimentally exposed to four different dengue virus isolates (two DENV-1 isolates, designated as DV1-26A and DV1-30A, and two DENV-3 isolates, designated as DV3-10A and DV3-14A). The use of outbred families for genetic mapping was inspired from a validated study design previously developed to investigate the genetic basis of natural mosquito resistance to human malaria parasites [28], [29]. To simulate a natural situation, we used naturally circulating virus isolates contemporaneous with the mosquitoes that were obtained from the serum of human patients. Their complete genome sequence confirmed that they were genetically distinct (Fig. S1). Genetic mapping was based on a set of microsatellite markers distributed across the *Ae. aegypti* genome, which consists of three chromosomes (Fig. S2). With one marker every 9.0 centiMorgans (cM) on average, marker density was entirely adequate for chromosomes 1 and 3. For chromosome 2, however, the paucity of valid and/or informative microsatellites resulted in poor coverage (1 marker every 23.4 cM). Therefore, we focus here on chromosomes 1 and 3 and provide mapping results for chromosome 2 as supplementary data.

Our genetic mapping strategy allowed us to detect genetic linkage (i.e., non-independence between marker allele segregation and phenotype) at two different levels for each marker. The first level measured the dependence of the phenotype on the mosquito genotype *regardless* of the virus isolate (i.e., the main host genotype effect across virus serotypes and isolates). The second level measured the dependence of the phenotype on the genotype *conditional* on the virus isolate (i.e., the interaction between virus isolate and mosquito genotype, a measure of G×G interactions). The methodology of our genetic survey (Fig. 1) differs significantly from conventional genetic mapping strategies because it does not rely on controlled crosses between inbred lines that represent extremes of a trait. Although conventional strategies maximize QTL detection power, they are not best suited to identify multi-allelic QTL naturally segregating within unmanipulated populations [30], [31]. The large number of progeny produced by a single parental pair of mosquitoes can be used as outbred families that are suitable for QTL mapping [28], [29].

Vector competence was scored 14 days after an infectious blood meal according to three distinct phenotypes: (*i*) the proportion of mosquitoes that developed a midgut infection, (*ii*) the proportion of infected mosquitoes in which infection disseminated from the midgut to head tissues, and (*iii*) the infectious viral titer in virus-infected head tissues. Midgut infection and viral dissemination are prerequisites for virus transmission by mosquito bite [32]. Infectious titer of disseminated virus is used as a proxy for transmission potential [33]. All phenotypes were based on detection of infectious virus by standard plaque assay.

## Results

**Figure 2:**
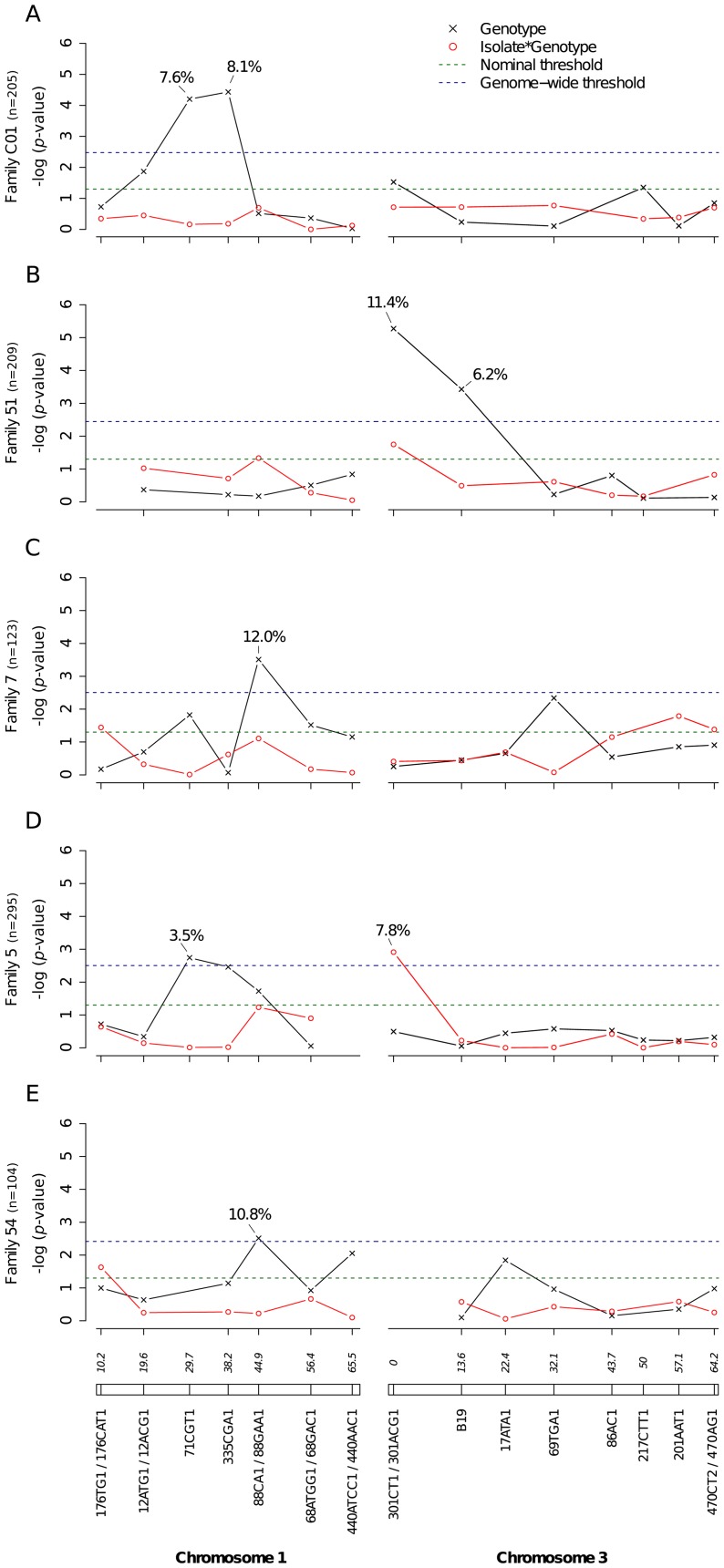
Genetic survey of *Ae. aegypti* loci associated with midgut infection. Midgut infection was assessed by the qualitative presence of infectious virus in individual mosquito bodies and analyzed as a binary trait. Nominal *p*-values are shown as a function of genetic marker positions (excluding uninformative markers) along chromosomes 1 and 3 (represented below the graphs with genetic distances in Kosambi cM). Dashed, horizontal lines indicate the nominal (green) and Bonferroni-corrected (blue) α = 0.05 statistical significance thresholds, respectively. The black line represents generalist effects (across virus serotypes and isolates) and the red line shows isolate-specific effects (genotype by isolate interactions). Above each significant marker, the percentage indicates the proportion of phenotypic variation explained by the marker, irrespective of other markers. Different graphs (A–E) correspond to different outbred mosquito families and therefore must be considered independently. Families do not carry the same amount of information with respect to QTL detection because the level of marker and QTL polymorphism varies among them.

A total of 2,084 *Ae. aegypti* females from nine independent isofemale families (mean sample size per family: 232; range: 104–403) were individually phenotyped and genotyped (Table S1). Five of the families yielded at least one QTL statistically significant at the genome-wide level for the midgut infection phenotype (Fig. 2). Significant linkage at the genome-wide level was detected on chromosome 1 at marker 71CGT1 (29.7 cM) in family C01 (genome-wide *p*-value = 9.44×10^−4^) and family 5 (*p* = 2.9×10^−2^), at marker 335CGA1 (38.2 cM) in family C01 (*p* = 5.55×10^−4^), and at marker 88CA1 (44.9 cM) in family 7 (*p* = 4.94×10^−3^) and family 54 (*p* = 4.0×10^−2^). Linkage was also detected on chromosome 3 at marker 301ACG1 (0.0 cM) in family 51 (*p* = 7.47×10^−5^) and at marker B19 (13.6 cM) in the same family (*p* = 5.22×10^−3^). The proportion of phenotypic variation explained by each significant marker ranged from 3.5% to 12.0%. Importantly, we also detected significant virus isolate-specific linkage on chromosome 3 at marker 301CT1 (0.0 cM) in family 5 (*p* = 1.95×10^−2^, Fig. 2D). In this family, the proportion of infected females varied significantly among 301CT1 genotypes, but the genotype-phenotype relationship differed between virus isolates (Fig. S3). This isolate-specific genotype-phenotype association is interpreted as a G×G interaction between the mosquito and the viral genomes. An underlying assumption is that the isolate effect is primarily driven by genetic differences among isolates. When the isolate was replaced by the corresponding blood meal titer in the analysis, the interaction effect was no longer statistically significant (*p* = 0.083), which ruled out that uncontrolled variation in infectious dose among virus isolates (Table S2) might have confounded our interpretation of the isolate effect.

**Figure 3:**
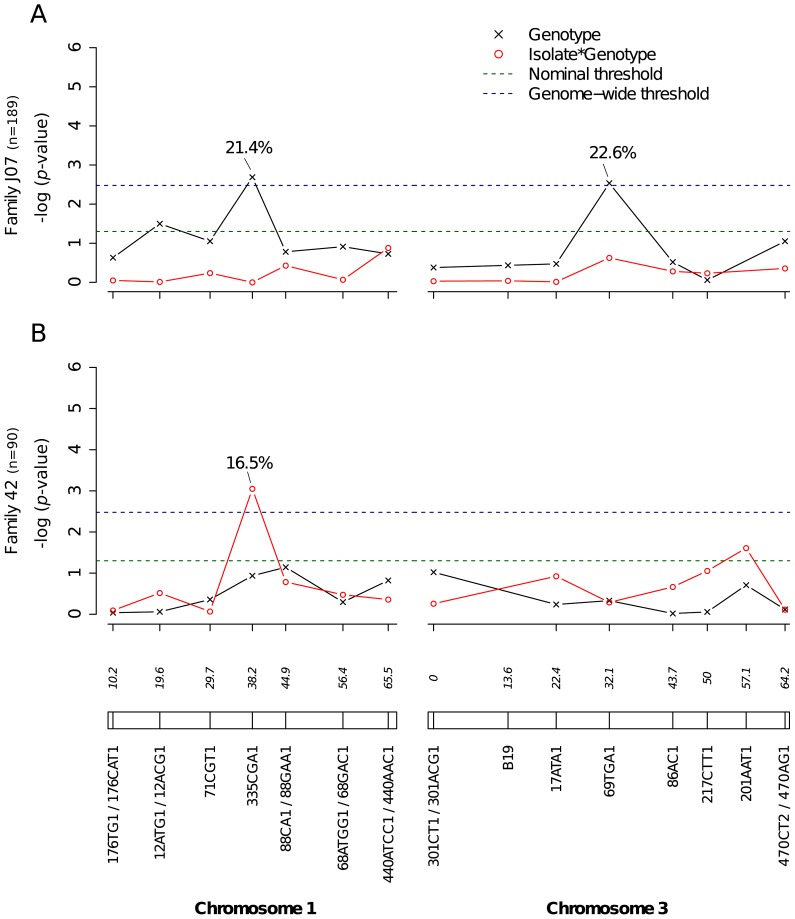
Genetic survey of *Ae. aegypti* loci associated with viral dissemination. Viral dissemination was assessed by the qualitative presence of infectious virus in individual mosquito heads and analyzed as a binary trait. This analysis only includes midgut-infected females. Different graphs (A–B) correspond to different outbred mosquito families and therefore must be considered independently. For details see Fig. 2 legend.

**Figure 4:**
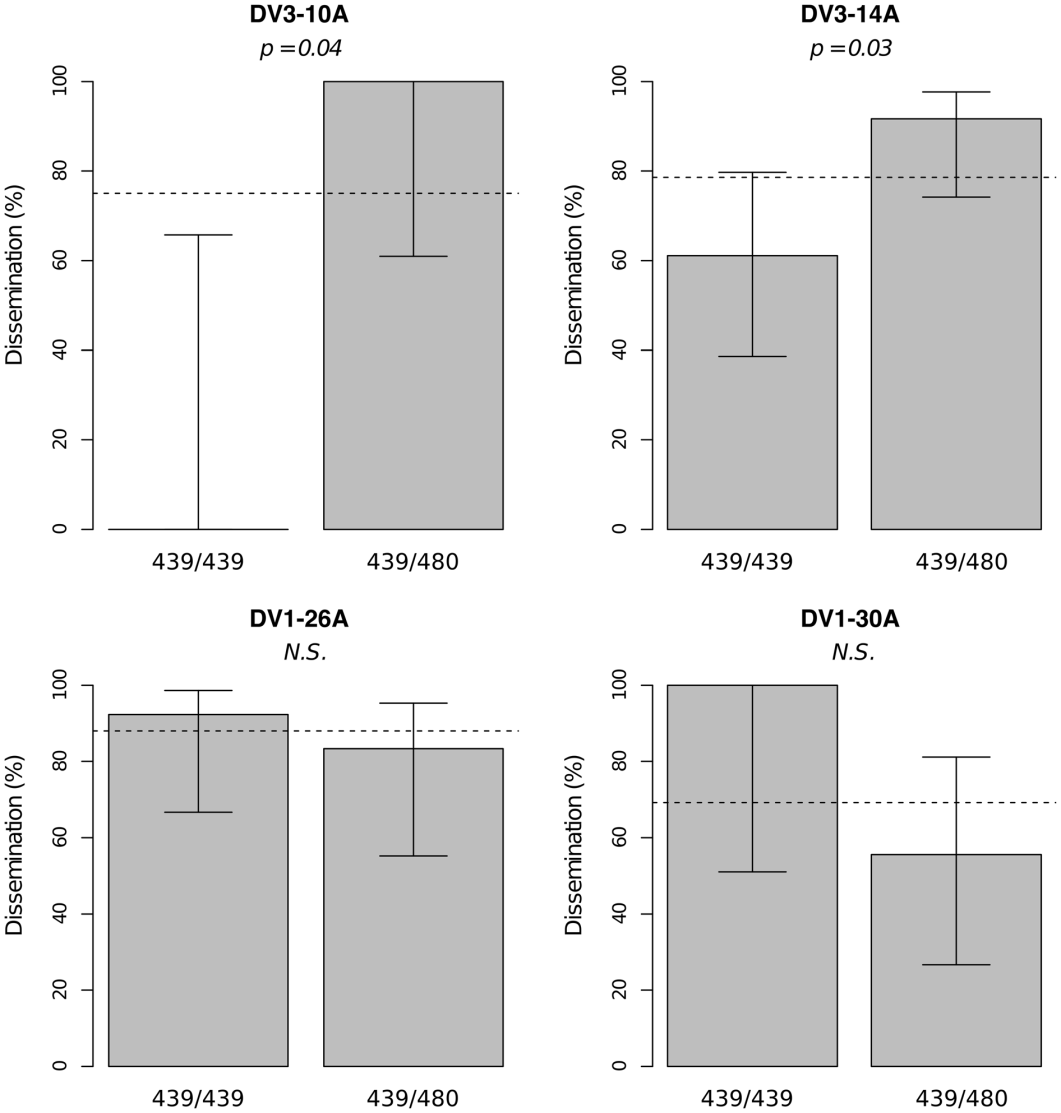
Isolate-specific association between marker 335CGA1 genotype and viral dissemination. Bars represent the percentage of midgut-infected females with a disseminated infection and their 95% confidence intervals for each genotype at the marker in isofemale family 42. The four panels correspond to the four dengue virus isolates tested in the study (DENV-3: DV3-10A, DV3-14A; DENV-1: DV1-26A, DV1-30A). Horizontal, dotted lines show the average percentage for each isolate. Only two marker genotypes (439/439 and 439/480) are present instead of the expected three genotypes because 335CGA1 is located at 38.2 cM on chromosome 1 in close proximity with the sex-determining locus (at 38.0 cM). Fig. S4 shows the inferred segregation of alleles at this marker. *P*-values above the graphs were obtained by pairwise comparison of proportions (Fisher's exact test; N.S. = not significant).

Significant linkage at the genome-wide level was detected in two of the nine families for the viral dissemination phenotype (Fig. 3). Linkage was significant on chromosome 1 at marker 335CGA1 (38.2 cM) in family J07 (*p* = 3.08×10^−2^) and family 42 (*p* = 3.1×10^−2^) and on chromosome 3 at marker 69TGA1 (32.1 cM) in family J07 (*p* = 4.4×10^−2^). The proportion of phenotypic variation explained by each significant marker ranged from 16.5% to 22.6%. Marker 335CGA1 on chromosome 1 was in linkage with the dissemination phenotype in two different families. In family J07 the marker effect was general across virus serotypes and isolates (Fig. 3A), whereas in family 42 it was isolate-specific (Fig. 3B). To verify that the isolate effect was not confounded with an effect of the infectious dose, we confirmed that the isolate by genotype interaction in family 42 was no longer statistically significant when the isolate was substituted by the blood meal titer (*p* = 0.287). For illustration, Fig. 4 shows the genotype-phenotype correlation for each virus isolate at marker 335CGA1 (the allele segregation pattern at this marker is shown in Fig. S4). Although marker genotype 439/439 confers protection against viral dissemination of isolates DV3-10A and DV3-14A, it does not have a detectable effect against isolates DV1-26A and DV1-30A. It is worth noting that because isolates DV3-10A and DV3-14A belong to DENV-3 whereas isolates DV1-26A and DV1-30A belong to DENV-1, in this particular case the effect could be serotype-specific rather than isolate-specific.

**Figure 5:**
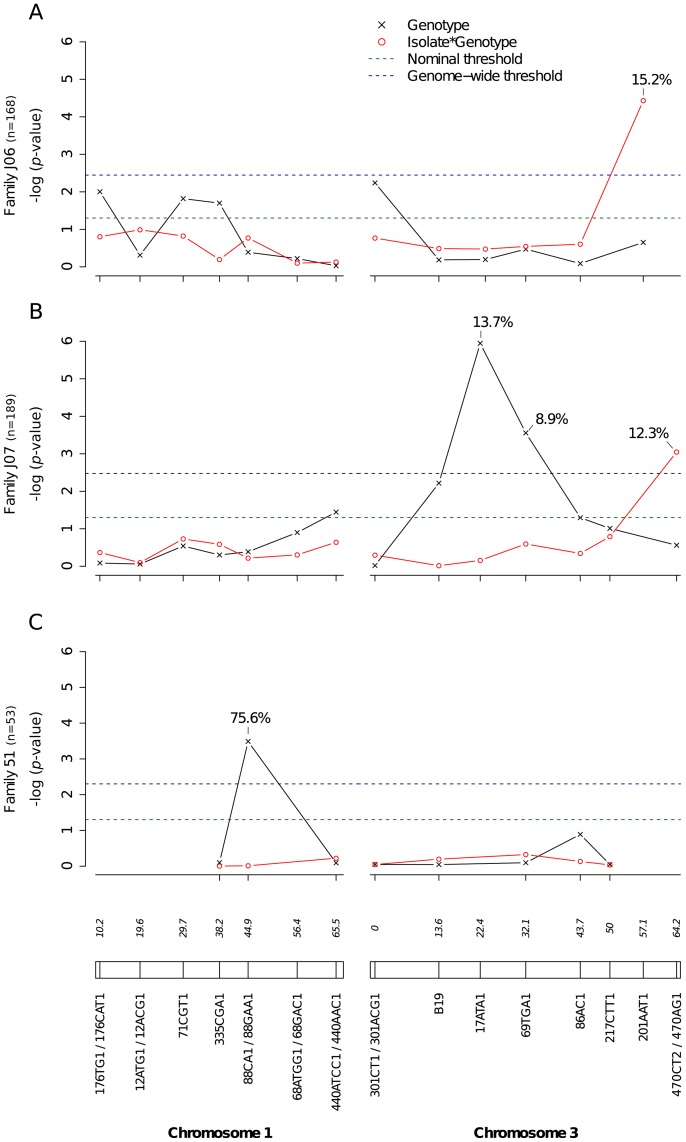
Genetic survey of *Ae. aegypti* loci associated with dissemination titer. Dissemination titer refers to the quantity of infectious virus in individual mosquito heads and was analyzed as a continuous trait. This analysis only includes females with a disseminated infection. Different graphs (A–C) correspond to different outbred mosquito families and therefore must be considered independently. For details see Fig. 2 legend.

**Figure 6:**
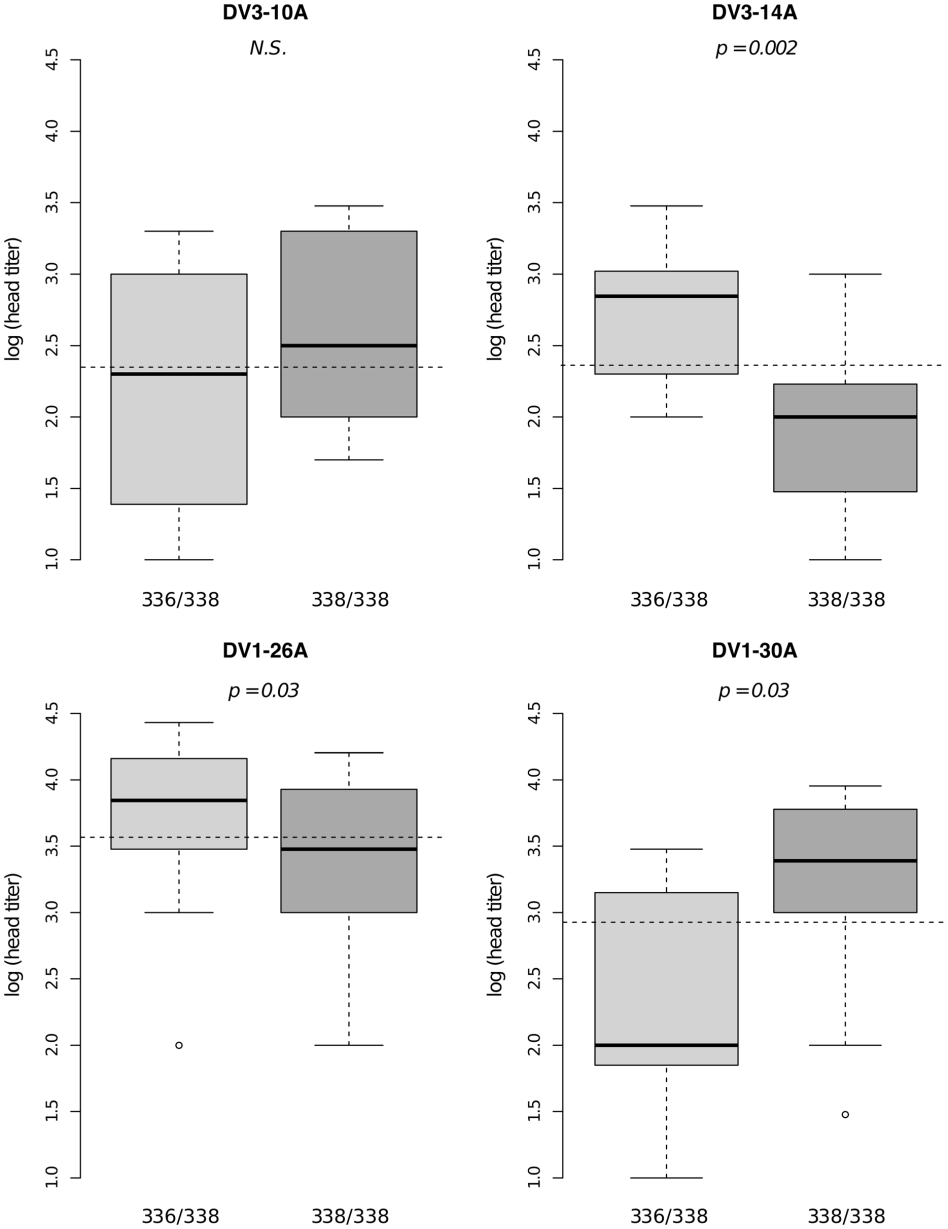
Isolate-specific association between marker 201AAT1 genotype and dissemination titer. Box plots represent the distribution of log-transformed head titers of *Ae. aegypti* females with a disseminated infection for each genotype at the marker in isofemale family J06. The four panels correspond to the four dengue virus isolates tested in the study (DENV-3: DV3-10A, DV3-14A; DENV-1: DV1-26A, DV1-30A). Horizontal, dotted lines show the average head titer for each isolate. Only two marker genotypes (336/338 and 338/338) are shown instead of the expected three because genotype 336/336 was not represented for one isolate and was therefore excluded from the analysis. *P*-values above the graphs were obtained by pairwise comparison of means (Student's t test; N.S. = not significant).

Significant linkage at the genome-wide level was detected in three of the nine families for the head titer phenotype (Fig. 5). Linkage was significant on chromosome 1 at marker 88CA1 (44.9 cM) in family 51 (*p* = 3.24×10^−3^). Linkage was also detected on chromosome 3 at marker 17ATA1 (22.4 cM) in family J07 (*p* = 1.70×10^−5^), at marker 69TGA1 (32.1 cM) in family J07 (*p* = 4.16×10^−3^), at marker 201AAT1 (57.1 cM) in family J06 (*p* = 5.18×10^−4^), and at marker 470CT2 (64.2 cM) in family J07 (*p* = 1.35×10^−2^). The proportion of phenotypic variation explained by each significant marker ranged from 8.9% to 75.6%. The genotype-phenotype association was isolate-specific at marker 201AAT1 in family J06 and at marker 470CT2 in family J07. Again, substituting the isolate by the corresponding blood meal titer ruled out a confounding effect of the infectious dose because the interaction was no longer statistically significant at marker 201AAT1 (*p* = 0.434) or at marker 470CT2 (*p* = 0.130). For illustration, Fig. 6 shows the genotype-phenotype correlation for each virus isolate at marker 201AAT1. Although marker genotype 338/338 confers protection against viral dissemination of isolates DV3-14A and DV1-26A, it results in increased head titer of isolate DV1-30A and no detectable effect against isolate DV3-10A. In this case the effect is truly isolate-specific (not serotype-specific) because isolates DV3-14A and DV1-26A (DENV-3 and DENV-1, respectively) share the same pattern whereas isolates DV1-26A and DV1-30A (both DENV-1) display opposite patterns. The isolate-specific genotype-phenotype correlation at marker 470CT2 is shown in Fig. S5.

Supporting information includes genetic mapping results for chromosome 2 (Fig. S6, S7, S8) and for families that did not produce any significant linkage (Fig. S9, S10, S11).

## Discussion

Our genetic survey demonstrates that G×G interactions between dengue viruses and mosquito vectors can be assigned to physical regions of the mosquito chromosomes. To the best of our knowledge, this is the first study to successfully locate G×G interactions in an invertebrate genome by marker-based genetic mapping. In agreement with the conclusions of a previous meta-analysis [17], we provide empirical evidence that the genetic architecture of host resistance depends on the pathogen strain. We establish the existence of tangible host genetic factors underlying G×G interactions in a natural invertebrate host-pathogen system. This is a critical first step towards their identification and characterization.

This study also provides important new information on the biology of dengue virus transmission in a natural situation. Phenotypic variation in the ability of field *Ae. aegypti* populations to serve as vectors of dengue viruses was previously observed [20], [21]. Genetic selection experiments [22] followed by QTL mapping studies using inbred selected lines [23]–[25] demonstrated a genetic basis for *Ae. aegypti* susceptibility to dengue virus infection and dissemination. Here, we provide direct evidence that a significant portion of natural phenotypic variation is genetically determined. We identify multiple genetic factors that control dengue susceptibility in a natural *Ae. aegypti* population, but show that the effect of these factors also depends on the virus genome.

Irrespective of G×G interactions, the relatively large proportion of phenotypic variation explained by the individual mosquito markers (up to 75.6%) reveals the existence of QTL with major effects. Interestingly, QTL underlying the midgut infection phenotype explained a smaller proportion of the phenotypic variation than QTL underlying the viral dissemination and dissemination titer phenotypes, suggesting a different genetic architecture. This hypothesis is supported by a similar observation in an earlier QTL mapping study [23]–[25]. Alternatively, this could be due to differences in marker informativeness or because exclusion of uninfected mosquitoes (on average, 57.5% of mosquitoes were uninfected in each family) for analysis of dissemination reduces the contribution of other QTL to overall phenotypic variation. Genetic linkage observed in different mosquito families could result from distinct loci or different allelic variants of the same locus. Based on the present data, we show that midgut infection by dengue viruses is controlled by at least two QTL in this wild *Ae. aegypti* population. In infected mosquitoes, viral dissemination from the midgut to secondary tissues is also controlled by two or more QTL. Infectious titer of disseminated virus, a proxy for transmission potential [33], is governed by three or more QTL.

Our mapping strategy relies on marker-by-marker tests and does not generate a confidence interval of the QTL location on the chromosomes. In other words, conventional techniques of interval mapping cannot be applied. Therefore, we cannot ascertain at this stage whether QTL identified on chromosomes 1 and 3 match those previously mapped for a DENV-2 strain in laboratory systems. On chromosome 1, a midgut infection QTL was previously identified at 19 cM [25] and a dissemination QTL at 31 cM [23]. On chromosome 3, a dissemination QTL was previously identified between 44 and 52 cM [23], [24]. No QTL was reported at the extremities of chromosome 3 in earlier studies. In the present study, significant linkage detected in the vicinity of the sex-determining locus (38.0 cM on chromosome 1) in four different families for the infection phenotype (Fig. 2A, 2C, 2D, 2E), in two families for the dissemination phenotype (Fig. 3A, 3B), and in one family for the head titer phenotype (Fig. 5C), could point to a major gene, or cluster of genes, controlling mosquito-virus interactions. Another important limitation of our marker-by-marker mapping strategy is that epistatic interactions between mosquito loci could not be measured. Intragenomic epistasis is a major component of the genetic architecture of quantitative traits [34], including host susceptibility to pathogens [17]. It is recognized as an essential determinant of the structure and evolution of complex genetic systems [35].

The main innovation of our study design was to explicitly account for viral genetic diversity in the genetic mapping of mosquito susceptibility loci. This allowed detection of both generalist and isolate-specific susceptibility loci. Several of the significant markers were in linkage with the phenotype independently of the virus isolate. Thus, the genetic basis of *Ae. aegypti* susceptibility to dengue viruses comprises a generalist component that is effective against diverse isolates, including isolates belonging to different serotypes. This result was previously unknown and gives hope to identify antiviral genes that confer a generalist protection against a diverse array of viruses. On the other hand, our genetic survey detected an isolate-specific component of the mosquito genetic basis for dengue susceptibility, which we interpret as G×G interactions between the vector and the virus. Markers associated with G×G interactions explained a significant proportion of phenotypic variation (from 7.8% to 16.5%). Identification of QTL associated with G×G interactions rules out the possibility that genetic specificity in this system is solely driven by environmentally inherited symbiotic microbiota that could have been confounded with the host genotype [16]. Note that this does exclude an indirect role of microbiota because the type of microbiota itself might be controlled by the host genotype.

It will be interesting to carry out fine-scale mapping experiments to identify the causal polymorphisms and their allelic profiles in the genomic regions where significant markers were found. An extension of the same protocol could be used to generate outbred isofemale lines beyond the F_2_/F_3_ generations to increase mapping resolution and locate candidate genes. Although several resistance mechanisms have been characterized in laboratory systems, mosquito genes underlying phenotypic variation in susceptibility to dengue viruses in nature have remained elusive. Leading candidates are genes known to be functionally involved in *Ae. aegypti* antiviral defense, including genes of the RNA interference (RNAi), JAK-STAT and Toll pathways [36]–[38]. A key gene of the RNAi pathway was recently associated with G×G interactions in this system [39]. The extremely low frequency (∼0.1%) of dengue virus infected *Ae. aegypti* in nature [40] and the relatively modest fitness cost of infection [41] make it unlikely that occasional challenge by dengue viruses is a strong enough selective pressure to drive the evolution of these genes. Rather, we speculate that their evolutionary dynamics are shaped by their concomitant role in the response to more prevalent pathogens in wild mosquito populations [42]. Conversely, natural selection of viruses that are able to evade or suppress resistance mechanisms is more likely to occur. Selection for enhanced transmission by mosquitoes has been proposed as a possible mechanism of adaptive evolution in dengue viruses [33].

Our results have at least two practical implications for the current development of novel strategies to interrupt virus transmission by genetically engineering resistant mosquitoes [43], [44]. First, the observation that *Ae. aegypti* vector competence for dengue viruses is controlled by multiple segregating QTL in a natural population suggests that such strategies may need to knock-down a larger number of genes than previously thought to confer complete resistance. Second, our discovery that the effect of several QTL is dengue virus serotype- and/or isolate-specific highlights the requirement for engineered resistance to be effective across all possible virus serotypes and strains encountered in nature.

In conclusion, our findings reinforce the idea that contributions from different genomes to the genetic architecture of ecological interactions cannot be fully disentangled because they depend on one another. By analogy with epistasis within the genome of a single organism, whereby the effect of a particular genotype on the phenotype depends on the genetic background, the direction and/or magnitude of the effect of host genes may depend on the pathogen genetic make-up. Like epistasis [45], [46], such G×G interactions between the genomes of two (or more) interacting organisms may constitute a significant component of the genetic architecture of complex traits resulting from ecological interactions. This may be true not only for antagonistically interacting organisms such as hosts and pathogens, but also for mutualistic interactions between, for example, animals and their gut microbiota or plants and their root microbiota [47], [48]. Accounting for the contribution of such genetic interactions between genomes will advance our understanding of the full genetic architecture of complex interaction traits in nature.

## Materials and Methods

### Mosquito families

Wild mosquito eggs were collected using ovitraps in several households in the Nhong Pling, Kon Tee, Mae Na Ree, Nhong Ping Kai, and Thep Na Korn subdistricts, Muang district, Kamphaeng Phet Province, Thailand, during May 2010 and February 2011. Kamphaeng Phet Province is an agrarian, sparsely populated area located approximately 350 km northwest of Bangkok where dengue is endemic and the four dengue virus serotypes co-circulate [49]. All collections were made in rural villages located within a localized area of less than 850 km^2^. F_0_ eggs were brought back to the AFRIMS laboratory in Bangkok and allowed to hatch in filtered tap water. F_0_ pupae were separated and allowed to emerge in individual vials. *Aedes aegypti* adults were identified by visual inspection.

Single F_0_ pairs consisting of one virgin male and one virgin female were allowed to mate for 2–3 days following emergence. To avoid that F_0_ parents were siblings from the same wild mother, the male and the female of each pair were chosen from different collection sites. Inseminated females were offered daily blood meals and allowed to lay eggs. Egg batches from a single female were merged to obtain a pool of F_1_ eggs. F_0_ males and females were saved for later DNA extraction and typing. F_2_ and F_3_ families were produced by mass sib-mating and collective oviposition from the F_1_ offspring. Although the mass-mating step reduces statistical power to detect genetic linkage because parental genetic information is partially lost, it is traded for a considerable increase in sample size [28]. A single *Ae. aegypti* pair can produce several thousands progeny per generation after as few as 2–3 generations in the laboratory.

F_1_ adults were allowed to emerge in the laboratory, mate randomly, and feed on defibrinated sheep blood (National Laboratory Animal Center, Mahidol University, Bangkok, Thailand) through a membrane feeding system. The F_2_ and F_3_ eggs were collected and stored on dry pieces of paper towel and maintained under high humidity no longer than 6 months.

Although most *Ae. aegypti* females are inseminated by a single male in nature [50], using single pairs of newly emerged mosquitoes instead of naturally inseminated females allowed us to genotype both F_0_ parents prior to phenotyping. Families are not equal in the information they bring to QTL detection. Only families with the highest proportion of polymorphic markers were retained for genetic mapping. The aim of choosing families was to maximize the number of informative (i.e., segregating) meiosis at both marker and susceptibility loci. Out of a total of 184 initial mating pairs, nine families were selected that had >3,000 F_2_/F_3_ eggs and >80% polymorphic markers.

### Virus isolates

Four low-passage dengue virus isolates (two DENV-1 and two DENV-3) were used to orally challenge mosquitoes in vector competence assays (Table S2). They derived from serum samples collected between March and July 2010 during routine surveillance for diagnostic public health testing at AFRIMS from clinically ill dengue patients attending Kamphaeng Phet Provincial Hospital. Phylogenetic analysis assigned the viruses to known lineages of DENV-1 and DENV-3 that were circulating in Southeast Asia in the previous years (Fig. S1). Each isolate was amplified twice in *Aedes albopictus* cells (C6/36, ATCC CRL-1660), which is the minimum required to obtain a viral titer sufficiently high to infect mosquitoes orally using an artificial blood meal. To prepare virus stock, 0.2 ml of human serum was inoculated onto 2-day-old confluent C6/36 cells in a 25-cm^2^ flask and incubated for 7 days at 28°C. The virus-infected cell culture supernatant was harvested and inoculated into a fresh flask of 2-day-old C6/36 cells for the second passage, of which supernatant was aliquoted and stored at −70°C.

### Virus sequencing and phylogenetic analysis

Viral genomic RNA was extracted from viral stock with the QIAamp viral RNA kit (Qiagen, Valencia, CA, USA). RT-PCR was performed using the SuperScript One-Step RT-PCR kit with platinum Taq polymerase (Invitrogen Life Technologies, Carlsbad, CA, USA) according to the manufacturer's recommendations, with a set of primers covering the entire genome (Table S3). RT-PCR products were purified by ultrafiltration. Sequencing reactions were performed using the Big Dye Terminator v1.1 cycle sequencing kit (Applied Biosystems, Foster City, CA, USA). Sequence chromatograms from both strands were obtained on an automated sequence analyzer ABI3730XL (Applied Biosystems). For sequence analysis, contig assembly and sequence alignments were performed using BioNumerics v6.5 (Applied-Maths, Sint-Martens-Latem, Belgium; www.applied-maths.com). Phylogenetic relationships were inferred using the maximum-likelihood method with the Tamura-Nei model implemented in MEGA v5 [51]. Reliability of nodes was assessed by bootstrap resampling with 1,000 replicates. The complete viral genome sequences were deposited to the GenBank database (accession numbers HG316481–HG316484).

### Experimental infections

*Ae. aegypti* females of the F_2_ or F_3_ generation were used in vector competence assays to score their relative susceptibility to the four low-passage dengue virus isolates. Experimental infections were run in three large experiments that involved different triplets of mosquito families (Table S1). F_2_/F_3_ eggs were hatched synchronously by placing them under low pressure for 30 min. Larvae were reared in 24×34×9 cm plastic trays filled with 2.0 liters of filtered tap water at a density of approximately 200 first instars per tray and fed a standard diet of approximately 1.0 g of fish food pellets (C.P. Hi Pro; Perfect Companion Group Co. Ltd., Bangkok, Thailand) per tray. Pupae were transferred to plastic screened 30×30×30 cm cages (Megaview Science Education Service Co. Ltd., Taichung, Taiwan) and adults were maintained on a diet of 10% sucrose. They were kept in an insectary at 28±1°C, under a relative humidity of 70–80% and a 12∶12 h light-dark cycle. The day before the oral challenge, females were transferred from the rearing cage to 1-pint feeding cups of ∼100 females.

Prior to experimental infections, 25-cm^2^ flasks of 2-day-old C6/36 cells were inoculated with a 1-ml aliquot from the viral stock and incubated for 45 min to 1 hour. At the end of the adsorption, 4.0 ml of maintenance medium were added and the cells were incubated at 35±1°C under 5% CO_2_ for 5 days. At day 5, 1.0 ml of heat-inactivated fetal bovine serum containing 15% of sodium bicarbonate 7.5% solution (HIFBS-NaHCO_3_) was added to the virus-infected cell culture supernatant, which was then harvested to prepare the infectious blood meal. The virus suspension was diluted 1∶3 or 1∶2 with RPMI 1640 medium containing 5% HIFBS and then mixed 1∶1 with defibrinated sheep blood (National Laboratory Animal Center). The infectious blood meal was placed in water-jacketed glass feeders maintained at a constant temperature of 37°C and covered with a piece of desalted porcine intestine. Four- to 7-day-old *Ae. aegypti* females deprived of sucrose and water for 24 h prior to blood feeding were offered an infectious blood meal for 30 min. Samples of the blood meals were saved for subsequent titration. Blood meal titers ranged from 2.0×10^4^ to 1.5×10^6^ plaque-forming units per ml (PFU/ml); the majority (83.3%) ranged between 1.0×10^5^ and 1.0×10^6^ PFU/ml (Table S2). Small differences in blood meal titers contribute to the isolate effect in the analysis, but we verified that it did not confound our interpretation (see below). After blood feeding, mosquitoes were briefly sedated with CO_2_ from dry ice, and fully engorged females were transferred to clean 1-pint paper cups. Unfed or partially fed females were discarded. Engorged females were maintained for 14 days at 28±1°C, under 70–80% relative humidity and a 12∶12 h light-dark cycle and provided cotton soaked with 10% sucrose *ad libitum*.

### Phenotypes

Vector competence was scored in the F_2_/F_3_ families at 14 days after the infectious blood meal according to three phenotypes: (*i*) midgut infection, (*ii*) viral dissemination from the midgut, and (*iii*) infectious titer in head tissues. Viral infection of midgut epithelial cells and subsequent dissemination to secondary tissues are two essential steps of dengue virus propagation in *Ae. aegypti*. Both events are prerequisites for virus transmission by mosquito bite and have been used to define a ‘midgut infection barrier’ and a ‘midgut escape barrier’ underlying *Ae. aegypti* susceptibility to dengue viruses [32]. These two vector competence indices were determined qualitatively (i.e., presence or absence of virus in mosquito bodies and heads, respectively). Although both phenotypes are binary traits (all-or-nothing), they are assumed to be consistent with a multifactorial basis and to result from continuous variation on an underlying (unobserved) scale. Infectious titer of virus disseminated to head tissues is strongly correlated with the probability to detect virus in saliva samples collected *in vitro* [33], and is therefore used as a proxy for transmission potential. Head titers were determined quantitatively by end-point titration.

Upon harvest, the head of each female was cut off on a chill table and placed individually in 500 µl of mosquito diluent (MD; RPMI 1640 medium with 10% HIFBS, 100 units/ml penicillin, 100 µg/ml streptomycin and 100 units/ml L-Glutamine). The remainder of the body (thorax and abdomen) was stored separately in 900 µl of MD with one 4.5 mm stainless steel bead in a 2-ml safe-lock tube. Samples were stored at −70°C until testing by plaque assay. They were quickly thawed in a water bath at 35±2°C and homogenized in a mixer mill (Qiagen) at 24 cycles/sec for 2 min. Four hundreds µl of each body homogenate were transferred into a new 1.5 ml safe-lock tube containing 400 µl of lysis buffer BQ1 (Macherey-Nagel, Düren, Germany) and stored at −20°C for DNA genotyping.

Infectious virus was detected and quantified by plaque assay performed in rhesus monkey kidney epithelial cells (LLC-MK_2_, ATCC CCL-7) as previously described [52]. Briefly, the homogenized body and head samples were filtered individually through a sterile, syringe-mounted 0.22-µm membrane. The samples were placed in an ice bath, 100 µl/well were inoculated onto a monolayer of 3-day-old LLC-MK_2_ cells in 24-well plates. The virus was adsorbed at room temperature (20–28°C) on a rocker platform for 90 min. The inoculum was removed and 0.5 ml/well of a first overlay of medium was added. The cells were incubated for 5 days at 35±1°C under 5±0.5% CO_2_. The cells were stained with a second overlay of medium containing 4% neutral red (Sigma Chemical Co., Perth, WA, USA). Mosquito infection and dissemination status was determined based on the presence of plaques in their body and head homogenates, respectively. Mosquito whose bodies were negative by plaque assay were considered uninfected, and their heads were not processed further. Head titer of infected bodies was determined by plaque assay of 1∶10 and 1∶100 dilutions of head homogenates.

### Genetic survey

QTL detection was performed in the outbred mosquito families using a set of 25 microsatellite markers broadly distributed across the genome (Fig. S2). Genetic position and PCR primers sequences for these markers were readily available from published literature [53], [54] with the exception of markers 210TTC1 and 14ATT1 that we developed (see below) in an attempt to increase chromosome 2 coverage. In our *Ae. aegypti* population, few existing chromosome 2 markers were valid and/or informative, and despite our efforts to find additional markers, coverage remained too low to provide a sufficient mapping density of markers. The paucity of unique sequences among supercontigs mapped to chromosome 2 made it extremely difficult to design primer pairs resulting in unique PCR products. Efforts are currently being made to develop alternative markers based on single nucleotide polymorphisms (SNPs). For each marker in the final map (Fig. S2), we verified that the pair of primers matched a unique supercontig of the unassembled *Ae. aegypti* genome [55], which in turn was anchored to the reference genetic map [56] by the co-presence of another marker with known genetic position that uniquely matched the same supercontig. The only exception is marker B19 that falls in an unmapped supercontig but was independently assigned to chromosome 3 by linkage analysis [53]. The 25 microsatellites represent 18 distinct genetic positions along the *Ae. aegypti* genome. Twenty-two of these microsatellites (15 genetic positions) are located on chromosomes 1 or 3. Based on an estimated genome size of 1,376 Mbp and a genetic size of 205 centiMorgans (cM), the relationship between physical and recombination distance is 6.71 Mbp/cM [55], [56]. Estimated genetic sizes of chromosomes 1 and 3 are 70.6 and 64.2 cM, respectively [56]. For these two chromosomes, adjacent markers in our genetic survey were separated by an average distance of 9.0 cM (60.3 Mbp). Thus, an unknown QTL was on average less than 4.5% recombination away from a marker.

The genetic survey was based on the analysis of outbred mosquito families at the F_2_ or F_3_ generation. Each mosquito family descended from a single pair of F_0_ parents collected in the field, providing an independent sample of up to four different alleles per locus from the original natural mosquito population. Based on the number of alleles present at the F_0_ generation, we verified at each marker that the correct number of genotypes was observed in the progeny. Three, six and ten different genotypes are expected in the progeny when F_0_ parents harbor two, three and four different alleles, respectively.

The originality of the strategy is to use families with incomplete pedigree information due to the mass-mating step [28]. Mosquitoes are classified according to their genotype so that identity by state (IBS) is used as a surrogate for identity by descent (IBD). Genetic linkage is not inferred from allele sharing proportions but from genotype-phenotype associations. Therefore, allele segregation in Mendelian proportions is not required by the study design. During mass mating and collective oviposition allele frequencies may be distorted because of random genetic drift or natural selection. Genetic drift is particularly likely to occur at the F_1_ generation because the number of reproducing adults is relatively small. Some genotypes could also be selected because they have a fitness advantage over other genotypes in insectary conditions. Departure from a neutral reproductive model may reduce the statistical power to detect marker-trait associations, but not the statistical significance of results. The same is true for null alleles or genotyping errors that would confound the observed genotypes. Our genetic model does not specify allelic codominance or recessivity. It simply compares genotypes (or groups of genotypes if a null allele segregates) regardless of their frequency.

Statistical power is also limited by the extent of heterozygosity in the family. There is no guarantee that every F_0_ parent is heterozygous both at a QTL and at a linked segregating marker, which is a prerequisite to generate a marker-trait association in the progeny. We maximized statistical power by genotyping F_0_ parents and choosing the most informative families (i.e., with >80% of markers being polymorphic) for phenotyping. In addition, the linkage phase between the marker and the QTL can vary in the progeny. This can reduce QTL detection power, if for example the same marker allele is associated with different QTL alleles in the F_0_ parents. Again, this would increase the probability to declare significant evidence against marker-trait association (i.e., in support of the null hypothesis) but not the statistical significance of results.

### Novel markers

Microsatellite markers 210TTC1 and 14ATT1 on chromosome 2 were developed as previously described [54]. Briefly, supercontig sequences containing genetic markers mapped to chromosome 2 were retrieved from VectorBase (http://aaegypti.vectorbase.org/) and submitted to the Tandem Repeats Finder program [57] using default parameters with the exception of a maximum period size of 3. For tandem repeats with a consistent motif and a repeat copy number <30, a ∼500 bp sequence encompassing the microsatellite was subjected to BLASTn analysis against the *Ae. aegypti* genome in VectorBase to verify their occurrence in single copy. PCR primers were designed in flanking sequences of selected microsatellites using Primer3 v0.4.0 [58], with an amplicon size target of 100–500 bp in length. The primer sequences were 5′-TCATTCCCAGTACCACACAAACG-3′ (forward) and 5′-ACTCGTTACTGGATGTGCTATCCC-3′ (reverse) for marker 14ATT1 and 5′-GAACGCGCTCGTAAGCGAGA-3′ (forward) and 5′-CACTGTGCGTTGGTTTCGGCT-3′ (reverse) for marker 210TTC1. Individual primer pairs were further subjected to BLASTn analysis to verify that they were predicted to amplify single copy sequences in the *Ae. aegypti* genome. PCR products were run by electrophoresis on 2% agarose gel to confirm that amplicons were unique.

### Microsatellite genotyping

Genomic DNA was extracted from mosquito homogenates using the NucleoSpin 96 Tissue Core Kit (Macherey-Nagel) and stored at −20°C until use. Genotyping of microsatellite repeats was performed by PCR amplification using fluorochrome-labeled forward primers (5′-FAM, 5′-HEX or 5′-ATTO550) (Eurofins MWG Operon, Ebersberg, Germany) to generate fluorescent PCR products. Primer pairs producing different amplicon sizes were assembled into multiplex groups of 4–6 markers. Amplification was performed in 25 µl volumes in Thermo-Fast 96-wells PCR plates (ABgene, Epsom, Surrey, UK) in a Veriti thermal cycler (Applied Biosystems). Each reaction contained 1× Taq buffer (50 mM KCl, 20 mM Tris pH 8.4) (Invitrogen Life Technologies), 1.5 mM MgCl_2_, 200 µM dNTPs (Invitrogen Life Technologies), 0.2 µM of each primer, 1 unit of Taq DNA polymerase (Invitrogen Life Technologies), and 2 µl of genomic DNA purified as described above. Thermocycling conditions were 5 min at 94°C, followed by 35 cycles of a 30-sec denaturation at 94°C, a 30-sec annealing at 50°C, and a 1-min extension at 72°C, followed by a 7-min final extension at 70°C. Multiplexed PCR products were examined by electrophoresis on 1% agarose gel and diluted 1∶10 in sterile water. Two µl of this dilution was added to 10 µl of Hi-Di Formamide (Applied Biosystems) containing 7.5% of 6-carboxy-X-rhodamine (ROX)-labeled Geneflo 625 size standards (EurX, Gdansk, Poland). Capillary electrophoresis of multiplexed PCR products was performed on a 3730xl DNA Analyser (Applied Biosystems). Sizes of microsatellite alleles were called and manually checked using the GeneMapper v4.0 software package (Applied Biosystems).

### Statistical analysis

Our approach is a combination of linkage and association analyses. Linkage analysis generally uses pedigrees to infer the location of a susceptibility locus based on coinheritance of the disease phenotype with genetic markers whose chromosomal location is known. Association analysis does not rely on pedigree structure but assumes that strong associations between marker alleles and disease phenotype in a population will be due to linkage, rather than chance. In association studies, IBD due to coancestry is inferred from IBS in the form of observed allelic associations. In the present study, linkage was inferred from IBS as in association studies. Tests of genotype-phenotype associations, however, were performed in sibships (single-generation families) at the at the F_2_ or F_3_ generation. In contrast with association studies performed at the population level, high linkage disequilibrium in the families strongly reduces the marker density required for the genetic mapping.

Genetic linkage was inferred from the significance of the genotype effect in a generalized linear model of the phenotype that included the factors mosquito genotype, virus isolate and their interaction as explanatory variables. Response variables were the three vector competence indices that we measured: (*i*) midgut infection status, (*ii*) viral dissemination status of midgut-infected mosquitoes, and (*iii*) head titer in mosquitoes with a disseminated infection. For binary phenotypes (infection and dissemination), the model was fitted with a binomial error structure and a logit link function (i.e., a logistic regression). For the continuous phenotype (head titer), the variable was log-transformed and the model was fitted with a normal error distribution and an identity link function (i.e., a linear regression). The model was fitted separately for each informative microsatellite marker in each mosquito family. Depending on the number of alleles of the marker, the factor genotype had from three to ten different categories, whereas the factor isolate always had four categories (i.e., the four isolates used in the study). If, due to random sampling effects in the progeny, one category of the genotype was not encountered in one or more categories of the isolate, this genotype category was excluded from the analysis so that the genotype by isolate interaction could be tested in the model. Depending on the marker, this could result in a different number of mosquitoes included in the analysis for the same family.

Statistical significance of the genotype effect or the genotype by isolate interaction effect in the above model was determined differently for binary (infection and dissemination) and continuous (head titer) variables. For binary phenotypes, statistical significance was tested with an analysis of deviance [59]. The deviance measures the unexplained variation of the data for a given model. The difference between the deviances of two models measures whether the two models fit the data differently. We first tested whether a model with the factors isolate and genotype fitted the data significantly better than a model with only the isolate (i.e., testing whether the genotype is a significant predictor of the phenotype). Then we tested whether a model with isolate, genotype and genotype by isolate interaction fitted the data better than the model with only the main effects of isolate and genotype (i.e., testing whether the interaction is a significant predictor of the phenotype). To estimate the proportion of variation explained by a significant factor we compared the mean deviance (deviance divided by the number of degrees of freedom) of the model including the factor and the mean deviance of the model without the factor. For the continuous phenotype, statistical significance was tested with an analysis of variance. To estimate the proportion of variation explained by a significant factor we followed the approach described above for the binary phenotypes. We compared the residual variance (sum of squares divided by the number of degrees of freedom) of the model including the factor and the residual variance of the model without the factor.

Because we performed multiple tests for each mosquito family, we used a Bonferroni correction of the *p*-values to ensure a genome-wide type I error of at most α = 0.05 (i.e., no more than 5% false positives overall). The genome-wide significance level of the test at each marker was α/N, where N is the number of informative markers tested in each family. A genotype-phenotype association was declared significant at the genome-wide level if the nominal *p*-value was smaller than α/N. When a significant genotype by isolate interaction was found, we verified that uncontrolled differences in the infectious titer of the artificial blood meal (Table S2) did not confound our interpretation of the factor isolate as an approximation of viral genetic identity. We performed an analysis based on the same model as previously but replacing the isolate by the corresponding blood meal titer (log-transformed). If the isolate effect were only due to differences in blood meal titer, we expect that the effect would remain statistically significant. Conversely, if the effect became insignificant, it would mean that the isolate effect resulted primarily from an effect of the viral genetic polymorphism rather than a simple effect of the infectious dose.

All statistical analyses were performed in the statistical environment R [60].

## Supporting Information

Figure S1 Phylogenetic relationships among dengue virus isolates. Maximum likelihood trees based on complete genome sequences are shown for DENV-1 (A) and DENV-3 (B). Bootstrap values (1,000 replications) are indicated at the major nodes. Horizontal branch lengths are drawn to scale, with scale bars representing the number of substitutions per site. Sequences are labeled with their accession number followed by the sampling year and the country of origin. Previously described dengue virus ‘genotypes’ (large phylogenetic clades) are indicated. The four virus isolates of the study are in bold font. The background set of sequences was retrieved from GenBank.
(TIFF)

Figure S2 Marker map. Microsatellites used for the genetic survey are indicated with their genetic position in Kosambi centiMorgans (cM). Chromosomes are drawn to scale according to the reference genetic map [56]. Most of the markers were readily available from the literature [53], [54]. Two additional markers indicated with an asterisk (*) were developed in this study. Genetic positions were determined by the co-presence within the same supercontig of the microsatellite and another marker with known genetic position from linkage data [55], with the exception of marker B19 that falls in an unmapped supercontig but was independently assigned to chromosome 3 by linkage analysis [53].
(TIFF)

Figure S3 Isolate-specific association between marker 301CT1 genotype and midgut infection. Bars represent the percentage of infected *Ae. aegypti* females and their 95% confidence intervals for each genotype at the marker. The four panels correspond to the four dengue virus isolates tested in the study. Horizontal, dotted lines show the average percentage for each isolate.
(TIFF)

Figure S4 Inferred segregation of marker 335CGA1 in isofemale family 42. Parental genotypes and the observed frequency of F_2_ genotypes were used to reconstruct the segregation history. Numbers 439 and 480 refer to the size of PCR amplicons used to genotype the microsatellite alleles. Expected genotype frequencies are shown at the F_1_ and F_2_ generations for both sexes. The red asterisk indicates co-segregation with the male allele of the sex-determining locus (38.0 cM) closely linked with the marker (38.2 cM) on chromosome 1. In the F_2_ generation, only females were phenotyped so that the male genotypes (hatched) were not represented. Observed frequency of genotypes at the F_2_ generation were 45.5% of 439/439 and 54.5% of 480/439.
(TIFF)

Figure S5 Isolate-specific association between marker 470CT2 genotype and dissemination titer. Box plots represent the distribution of log-transformed head titers of *Ae. aegypti* females with a disseminated infection for each genotype at the marker. The four panels correspond to the four dengue virus isolates tested in the study. Horizontal, dotted lines show the average head titer for each isolate. Only four marker genotypes are shown instead of the expected six because two genotypes were not represented in all isolates and were therefore excluded from the analysis.
(TIFF)

Figure S6 Genetic survey for *Ae. aegypti* chromosome 2 loci associated with midgut infection. Nominal *p*-values are shown as a function of genetic marker positions (excluding uninformative markers) along chromosome 2 (represented below the graphs with genetic distances in Kosambi cM) in outbred mosquito families shown in Fig. 2. Dashed, horizontal lines indicate the nominal (green) and Bonferroni-corrected (blue) α = 0.05 statistical significance thresholds, respectively. The black line represents generalist genotype-phenotype associations (across virus serotypes and isolates) and the red line shows isolate-specific associations (genotype by isolate interactions). Different graphs (A–E) correspond to different mosquito families.
(TIFF)

Figure S7 Genetic survey for *Ae. aegypti* chromosome 2 loci associated with viral dissemination. Nominal *p*-values are shown as a function of genetic marker positions (excluding uninformative markers) along chromosome 2 (represented below the graphs with genetic distances in Kosambi cM) in outbred mosquito families shown in Fig. 3. Dashed, horizontal lines indicate the nominal (green) and Bonferroni-corrected (blue) α = 0.05 statistical significance thresholds, respectively. The black line represents generalist genotype-phenotype associations (across virus serotypes and isolates) and the red line shows isolate-specific associations (genotype by isolate interactions). Different graphs (A–B) correspond to different mosquito families.
(TIFF)

Figure S8 Genetic survey for *Ae. aegypti* chromosome 2 loci associated with head titer. Nominal *p*-values are shown as a function of genetic marker positions (excluding uninformative markers) along chromosome 2 (represented below the graphs with genetic distances in Kosambi cM) in outbred mosquito families shown in Fig. 5. Dashed, horizontal lines indicate the nominal (green) and Bonferroni-corrected (blue) α = 0.05 statistical significance thresholds, respectively. The black line represents generalist genotype-phenotype associations (across virus serotypes and isolates) and the red line shows isolate-specific associations (genotype by isolate interactions). Different graphs (A–C) correspond to different mosquito families.
(TIFF)

Figure S9 *Ae. aegypti* families with no significant locus associated with midgut infection. Nominal *p*-values are shown as a function of genetic marker positions (excluding uninformative markers) along the three chromosomes (represented below the graphs with genetic distances in Kosambi cM). Dashed, horizontal lines indicate the nominal (green) and Bonferroni-corrected (blue) α = 0.05 statistical significance thresholds, respectively. The black line represents generalist effects (across virus serotypes and isolates) and the red line shows isolate-specific effects (genotype by isolate interactions). Different graphs (A–D) correspond to different mosquito families.
(TIFF)

Figure S10 *Ae. aegypti* families with no significant locus associated with viral dissemination. Nominal *p*-values are shown as a function of genetic marker positions (excluding uninformative markers) along the three chromosomes (represented below the graphs with genetic distances in Kosambi cM). Dashed, horizontal lines indicate the nominal (green) and Bonferroni-corrected (blue) α = 0.05 statistical significance thresholds, respectively. The black line represents generalist effects (across virus serotypes and isolates) and the red line shows isolate-specific effects (genotype by isolate interactions). Different graphs (A–G) correspond to different mosquito families.
(TIFF)

Figure S11 *Ae. aegypti* families with no significant locus associated with head titer. Nominal *p*-values are shown as a function of genetic marker positions (excluding uninformative markers) along the three chromosomes (represented below the graphs with genetic distances in Kosambi cM). Dashed, horizontal lines indicate the nominal (green) and Bonferroni-corrected (blue) α = 0.05 statistical significance thresholds, respectively. The black line represents generalist effects (across virus serotypes and isolates) and the red line shows isolate-specific effects (genotype by isolate interactions). Different graphs (A–E) correspond to different mosquito families. Note that this analysis could not be performed for family 40 because the number of females with a disseminated infection was to small to support the statistical model.
(TIFF)

Table S1 Summary of raw vector competence data. For each pair of mosquito family and virus isolate, the number of mosquitoes, the number of informative markers, the percentage of mosquitoes with a midgut infection, the percentage of infected mosquitoes with a disseminated infection and the log-transformed mean viral titer (± standard deviation) in infected head tissues are indicated. In each experiment a different triplet of mosquito families at the F_2_ or F_3_ generation were simultaneously challenged with the four isolates.
(DOC)

Table S2 Description of virus isolates. The date of collection, serotype, number of passages in C6/36 cells, and measured infectious titers in the artificial infectious blood meals (in plaque-forming units per ml) are indicated. In each experiment a different triplet of mosquito families at the F_2_ (Experiment 2) or F_3_ generation (Experiments 1 and 3) were simultaneously challenged with the four isolates.
(DOC)

Table S3 Primers used for virus sequencing. Nucleotide positions and primer sequences are shown for each of the overlapping amplicons covering the viral genome.
(DOC)
