# Supplementary figures and images for: Genetic Mapping of Specific Interactions between Aedes aegypti Mosquitoes and Dengue Viruses
Source: PLoS Genet. 2013 Aug 1;9(8):e1003621. doi: 10.1371/journal.pgen.1003621 (PMC3731226; doi:10.1371/journal.pgen.1003621)

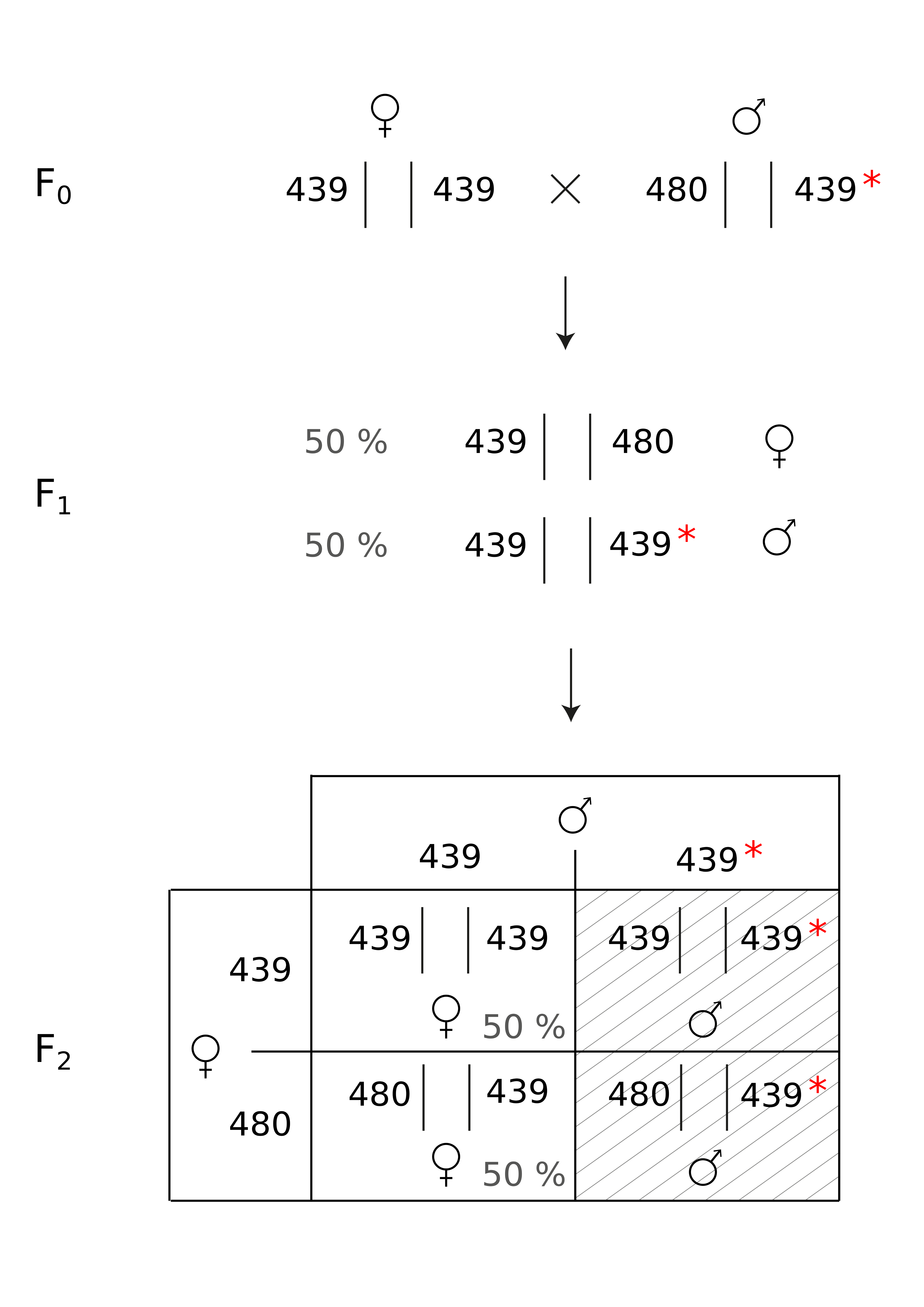

Supplement: Figure S4 — Inferred segregation of marker 335CGA1 in isofemale family 42. Parental genotypes and the observed frequency of F2 genotypes were used to reconstruct the segregation history. Numbers 439 and 480 refer to the size of PCR amplicons used to genotype the microsatellite alleles. Expected genotype frequencies are shown at the F1 and F2 generations for both sexes. The red asterisk indicates co-segregation with the male allele of the sex-determining locus (38.0 cM) closely linked with the marker (38.2 cM) on chromosome 1. In the F2 generation, only females were phenotyped so that the male genotypes (hatched) were not represented. Observed frequency of genotypes at the F2 generation were 45.5% of 439/439 and 54.5% of 480/439. (TIFF) [file pgen.1003621.s004.tiff]

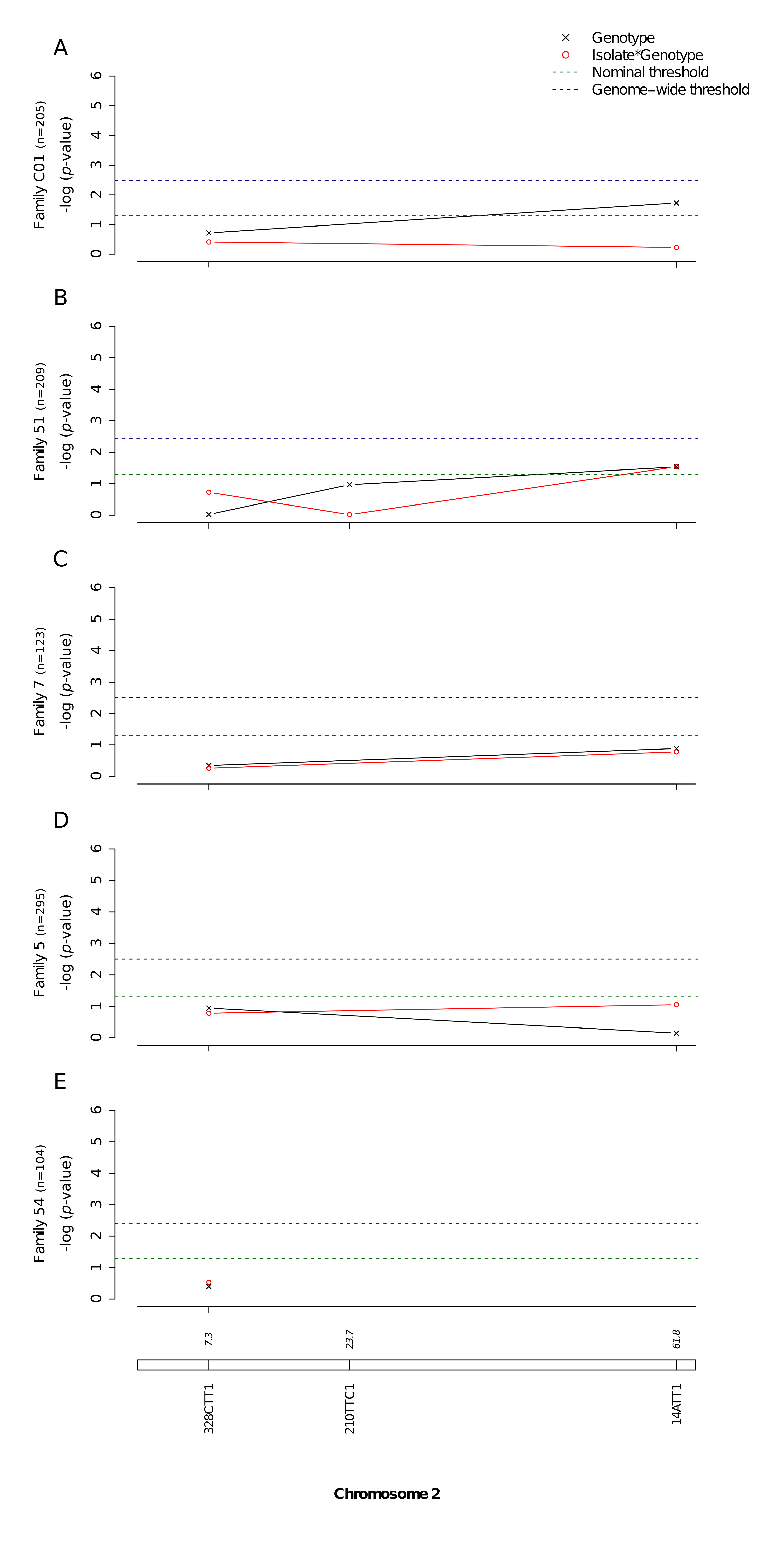

Supplement: Figure S6 — Genetic survey for Ae. aegypti chromosome 2 loci associated with midgut infection. Nominal p-values are shown as a function of genetic marker positions (excluding uninformative markers) along chromosome 2 (represented below the graphs with genetic distances in Kosambi cM) in outbred mosquito families shown in Fig. 2. Dashed, horizontal lines indicate the nominal (green) and Bonferroni-corrected (blue) α = 0.05 statistical significance thresholds, respectively. The black line represents generalist genotype-phenotype associations (across virus serotypes and isolates) and the red line shows isolate-specific associations (genotype by isolate interactions). Different graphs (A–E) correspond to different mosquito families. (TIFF) [file pgen.1003621.s006.tiff]

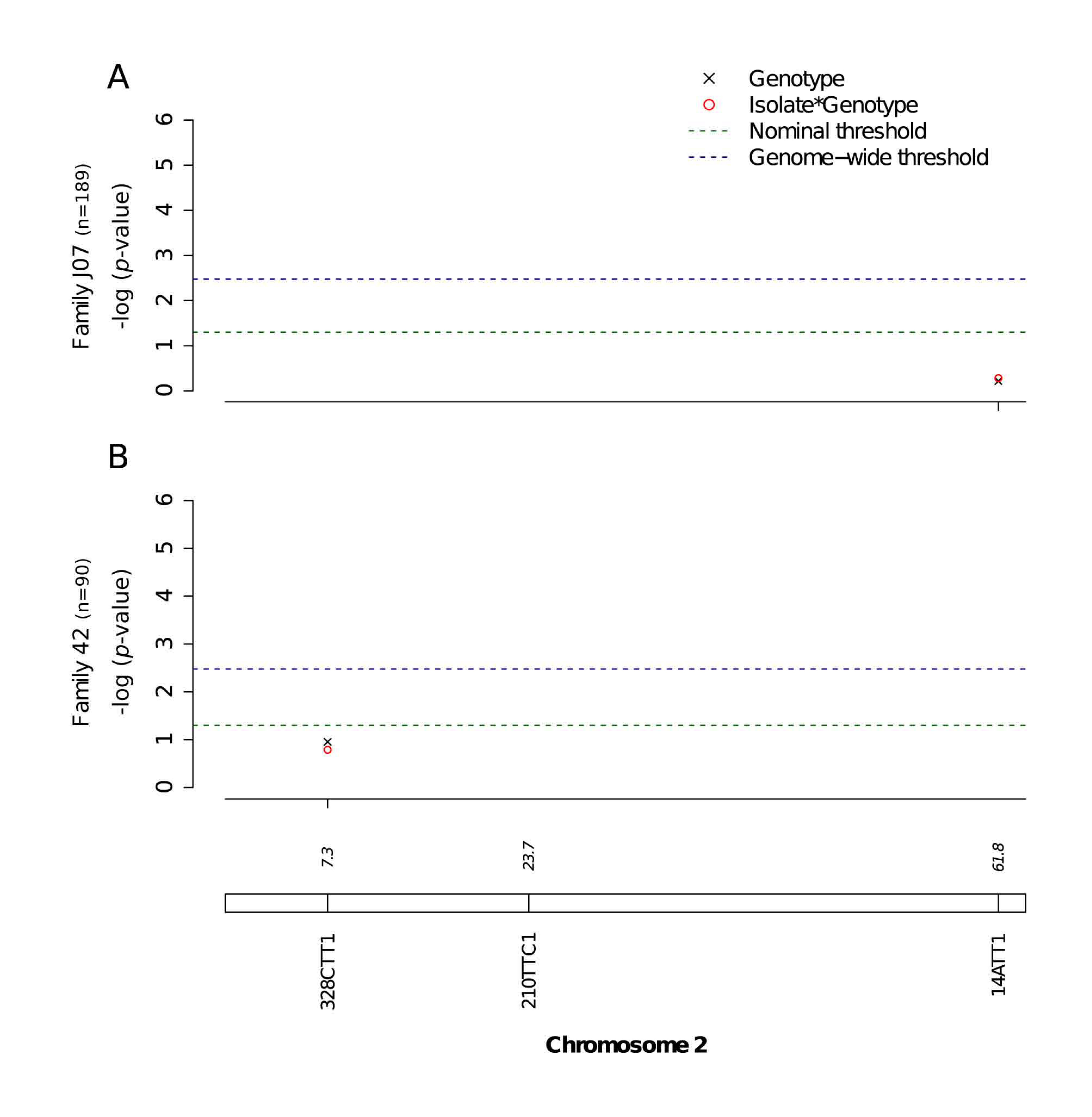

Supplement: Figure S7 — Genetic survey for Ae. aegypti chromosome 2 loci associated with viral dissemination. Nominal p-values are shown as a function of genetic marker positions (excluding uninformative markers) along chromosome 2 (represented below the graphs with genetic distances in Kosambi cM) in outbred mosquito families shown in Fig. 3. Dashed, horizontal lines indicate the nominal (green) and Bonferroni-corrected (blue) α = 0.05 statistical significance thresholds, respectively. The black line represents generalist genotype-phenotype associations (across virus serotypes and isolates) and the red line shows isolate-specific associations (genotype by isolate interactions). Different graphs (A–B) correspond to different mosquito families. (TIFF) [file pgen.1003621.s007.tiff]

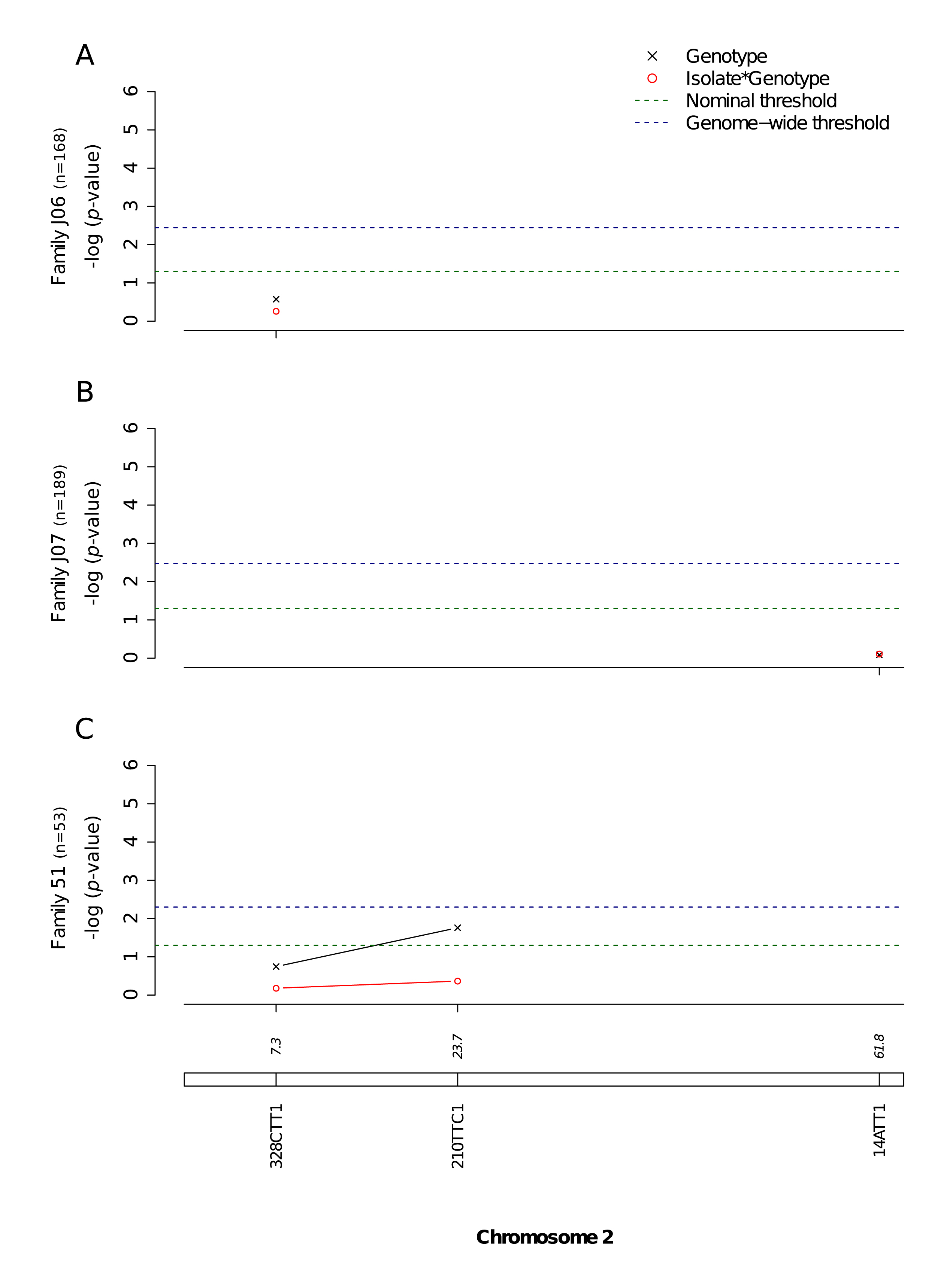

Supplement: Figure S8 — Genetic survey for Ae. aegypti chromosome 2 loci associated with head titer. Nominal p-values are shown as a function of genetic marker positions (excluding uninformative markers) along chromosome 2 (represented below the graphs with genetic distances in Kosambi cM) in outbred mosquito families shown in Fig. 5. Dashed, horizontal lines indicate the nominal (green) and Bonferroni-corrected (blue) α = 0.05 statistical significance thresholds, respectively. The black line represents generalist genotype-phenotype associations (across virus serotypes and isolates) and the red line shows isolate-specific associations (genotype by isolate interactions). Different graphs (A–C) correspond to different mosquito families. (TIFF) [file pgen.1003621.s008.tiff]

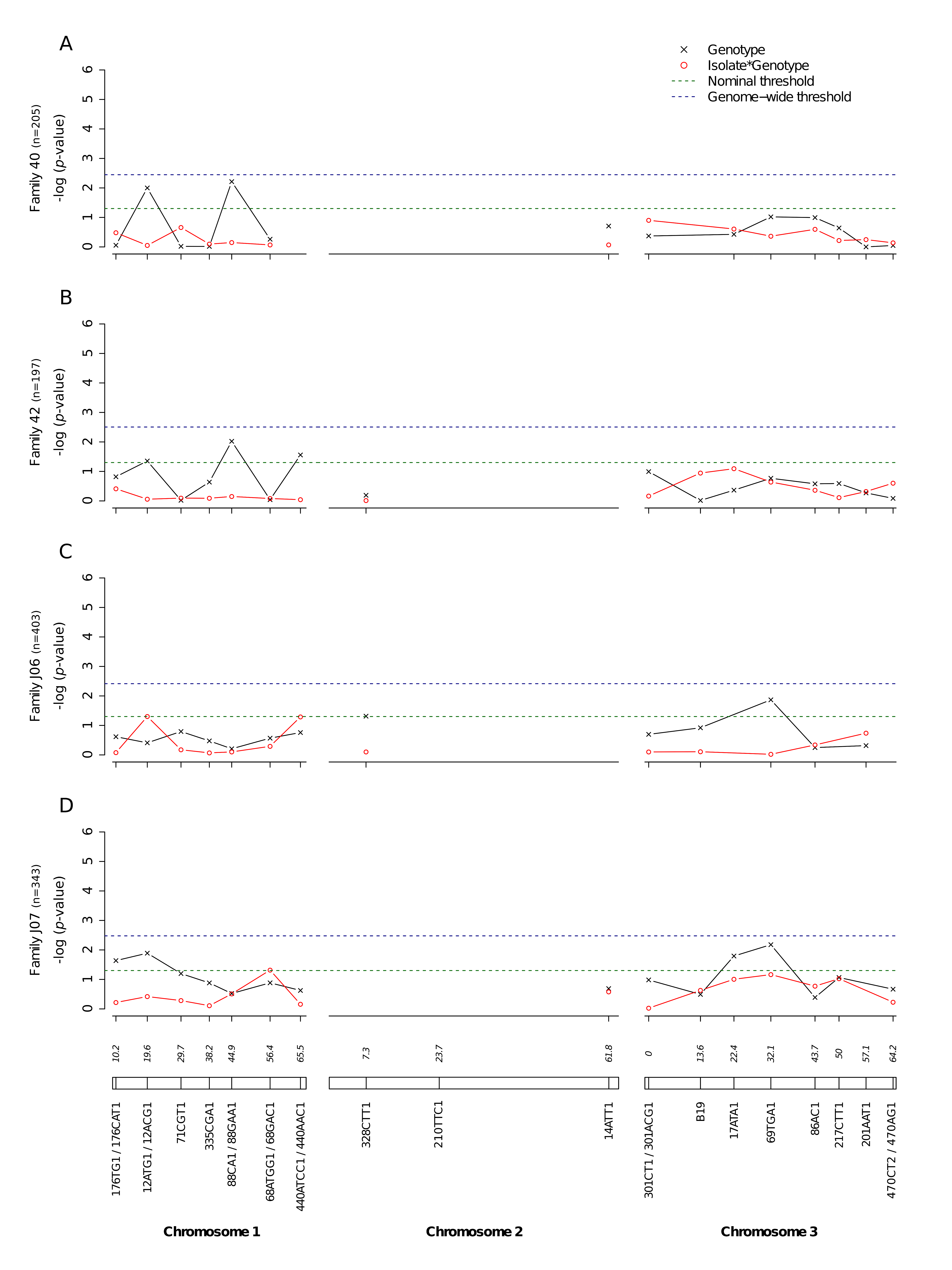

Supplement: Figure S9 — Ae. aegypti families with no significant locus associated with midgut infection. Nominal p-values are shown as a function of genetic marker positions (excluding uninformative markers) along the three chromosomes (represented below the graphs with genetic distances in Kosambi cM). Dashed, horizontal lines indicate the nominal (green) and Bonferroni-corrected (blue) α = 0.05 statistical significance thresholds, respectively. The black line represents generalist effects (across virus serotypes and isolates) and the red line shows isolate-specific effects (genotype by isolate interactions). Different graphs (A–D) correspond to different mosquito families. (TIFF) [file pgen.1003621.s009.tiff]

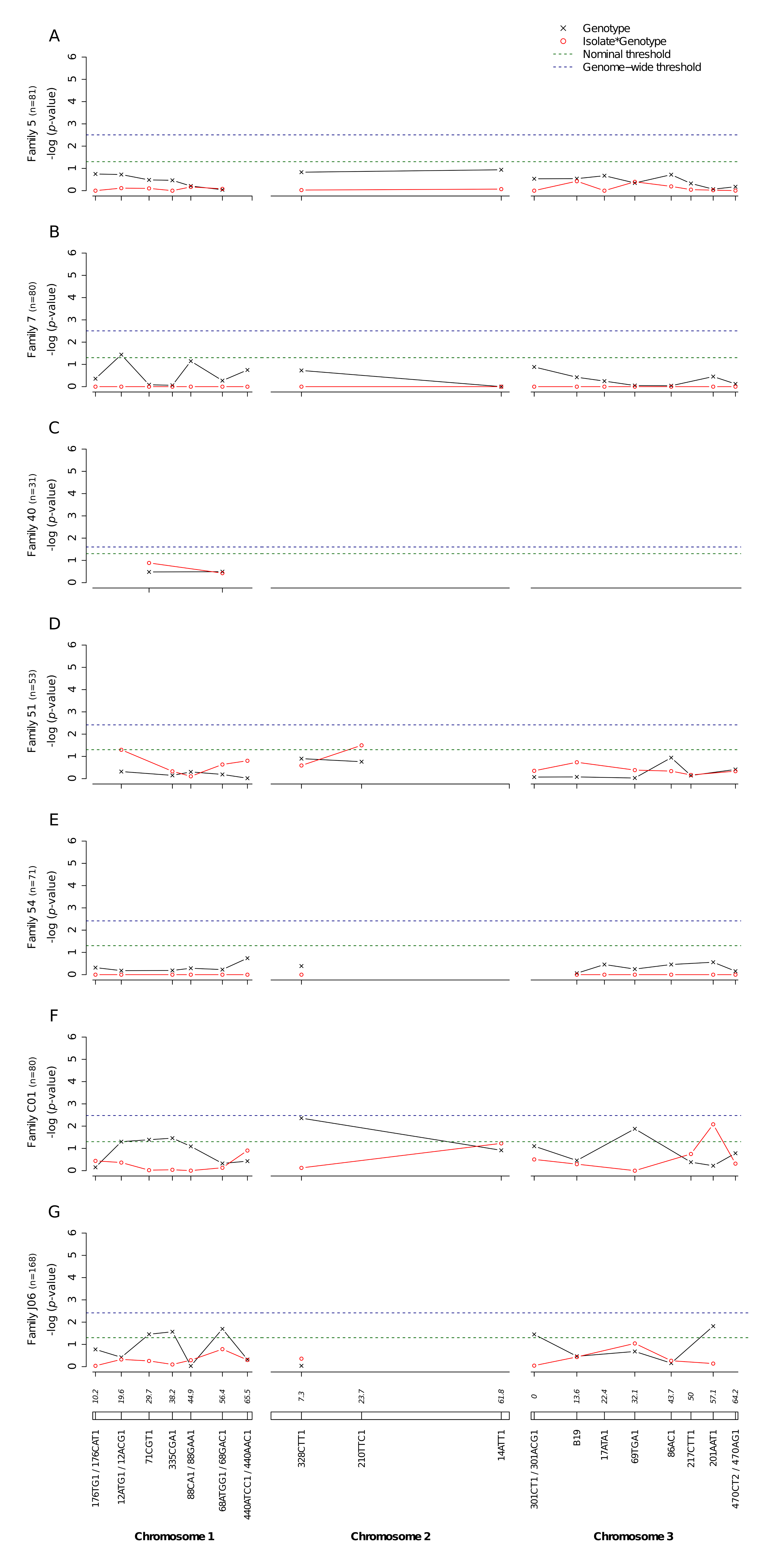

Supplement: Figure S10 — Ae. aegypti families with no significant locus associated with viral dissemination. Nominal p-values are shown as a function of genetic marker positions (excluding uninformative markers) along the three chromosomes (represented below the graphs with genetic distances in Kosambi cM). Dashed, horizontal lines indicate the nominal (green) and Bonferroni-corrected (blue) α = 0.05 statistical significance thresholds, respectively. The black line represents generalist effects (across virus serotypes and isolates) and the red line shows isolate-specific effects (genotype by isolate interactions). Different graphs (A–G) correspond to different mosquito families. (TIFF) [file pgen.1003621.s010.tiff]

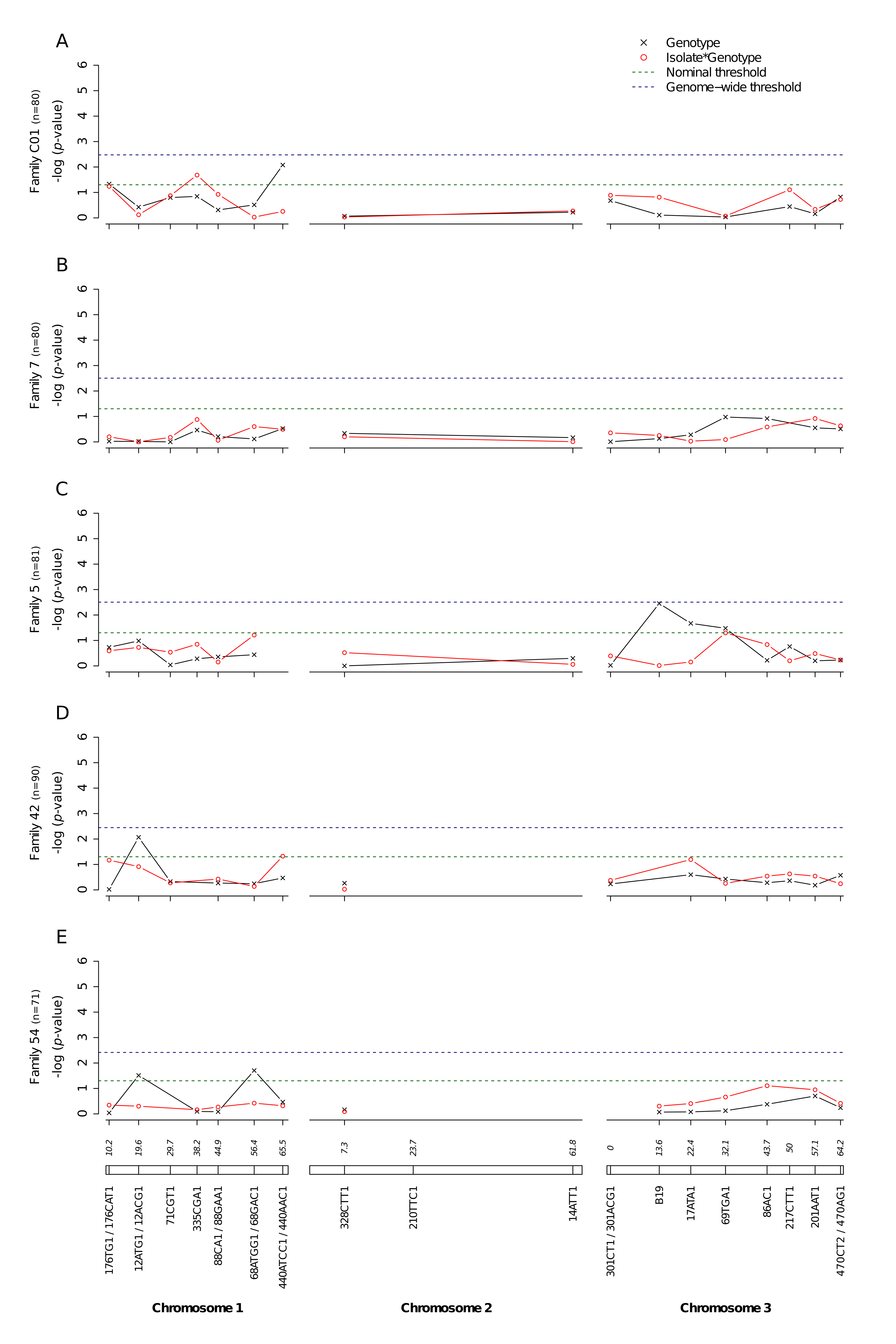

Supplement: Figure S11 — Ae. aegypti families with no significant locus associated with head titer. Nominal p-values are shown as a function of genetic marker positions (excluding uninformative markers) along the three chromosomes (represented below the graphs with genetic distances in Kosambi cM). Dashed, horizontal lines indicate the nominal (green) and Bonferroni-corrected (blue) α = 0.05 statistical significance thresholds, respectively. The black line represents generalist effects (across virus serotypes and isolates) and the red line shows isolate-specific effects (genotype by isolate interactions). Different graphs (A–E) correspond to different mosquito families. Note that this analysis could not be performed for family 40 because the number of females with a disseminated infection was to small to support the statistical model. (TIFF) [file pgen.1003621.s011.tiff]
