# Supplementary material for: Genetic Mapping of Specific Interactions between Aedes aegypti Mosquitoes and Dengue Viruses
Source: PLoS Genet. 2013 Aug 1;9(8):e1003621. doi: 10.1371/journal.pgen.1003621 (PMC3731226; doi:10.1371/journal.pgen.1003621)
Supplement: Table S1 — Summary of raw vector competence data. For each pair of mosquito family and virus isolate, the number of mosquitoes, the number of informative markers, the percentage of mosquitoes with a midgut infection, the percentage of infected mosquitoes with a disseminated infection and the log-transformed mean viral titer (± standard deviation) in infected head tissues are indicated. In each experiment a different triplet of mosquito families at the F2 or F3 generation were simultaneously challenged with the four isolates. (DOC) [file pgen.1003621.s012.doc]

**Table S1.** **Summary of raw vector competence data.** For each pair of mosquito family and virus isolate, the number of mosquitoes, the number of informative markers, the percentage of mosquitoes with a midgut infection, the percentage of infected mosquitoes with a disseminated infection and the log-transformed mean viral titer (± standard deviation) in infected head tissues are indicated. In each experiment a different triplet of mosquito families at the F_2_ or F_3_ generation were simultaneously challenged with the four isolates.

|  |  |  | | |  | |  | | | Midgut infection (%) | | | | Viral dissemination (%) | | | | Log head titer (mean±SD) | | | |
| --- | --- | --- | --- | --- | --- | --- | --- | --- | --- | --- | --- | --- | --- | --- | --- | --- | --- | --- | --- | --- | --- |
|  | Mosquito family ID | | Sample size by virus isolate | | | | | | Inform.  markers | Virus isolates | | | | Virus isolates | | | | Virus isolates | | | |
|  |  |  | Serotype 3 | | | Serotype 1 | | |  | Serotype 3 | | Serotype 1 | | Serotype 3 | | Serotype 1 | | Serotype 3 | | Serotype 1 | |
|  |  |  | 10A | 14A | | 26A | | 30A |  | 10A | 14A | 26A | 30A | 10A | 14A | 26A | 30A | 10A | 14A | 26A | 30A |
| Exp.1  (F_3_) | C01 | | 48 | 58 | | 55 | | 44 | 15 | 18.8 | 22.4 | 74.5 | 38.6 | 88.9 | 76.9 | 95.1 | 82.3 | 2.1±1.0 | 2.8±0.7 | 3.2±0.6 | 2.4±0.9 |
|  | J06 | | 95 | 116 | | 94 | | 98 | 13 | 27.4 | 35.3 | 71.3 | 34.7 | 65.4 | 70.7 | 98.5 | 85.3 | 2.3±0.8 | 2.4±0.7 | 3.6±0.6 | 2.9±0.9 |
|  | J07 | | 63 | 105 | | 108 | | 67 | 15 | 42.9 | 36.2 | 88.0 | 43.3 | 92.6 | 100 | 98.9 | 82.8 | 2.5±0.8 | 2.7±0.7 | 3.4±0.7 | 3.2±0.5 |
| Exp.2  (F_2_) | 40 | | 53 | 66 | | 39 | | 47 | 14 | 34.0 | 12.1 | 7.7 | 4.2 | 55.6 | 50.0 | 66.7 | 0.0 | 1.7±1.0 | 2.6±0.9 | 3.6±0.5 | n.a. |
|  | 42 | | 31 | 92 | | 37 | | 37 | 16 | 25.8 | 46.7 | 67.6 | 37.8 | 75.0 | 79.0 | 88.0 | 71.4 | 2.1±1.0 | 2.9±0.7 | 2.8±0.7 | 2.9±0.7 |
|  | 51 | | 55 | 46 | | 42 | | 66 | 14 | 16.3 | 19.6 | 40.5 | 27.3 | 55.6 | 55.6 | 82.3 | 77.8 | 2.1±0.6 | 2.1±0.7 | 2.2±0.6 | 2.0±0.8 |
| Exp.3  (F_3_) | 5 | | 90 | 64 | | 57 | | 84 | 16 | 11.1 | 53.1 | 40.4 | 16.7 | 80.0 | 88.2 | 100 | 100 | 2.8±0.7 | 2.4±0.6 | 2.9±0.7 | 2.9±0.6 |
|  | 7 | | 46 | 30 | | 27 | | 20 | 16 | 58.7 | 70.0 | 74.1 | 60.0 | 92.6 | 100 | 100 | 100 | 3.0±0.6 | 2.4±0.6 | 3.9±0.6 | 3.4±0.6 |
|  | 54 | | 18 | 21 | | 37 | | 28 | 13 | 50.0 | 66.7 | 83.8 | 60.7 | 88.9 | 100 | 100 | 100 | 2.9±0.4 | 2.1±0.7 | 3.1±0.6 | 2.7±0.6 |
