# Supplementary material for: Genetic Mapping of Specific Interactions between Aedes aegypti Mosquitoes and Dengue Viruses
Source: PLoS Genet. 2013 Aug 1;9(8):e1003621. doi: 10.1371/journal.pgen.1003621 (PMC3731226; doi:10.1371/journal.pgen.1003621)
Supplement: Table S2 — Description of virus isolates. The date of collection, serotype, number of passages in C6/36 cells, and measured infectious titers in the artificial infectious blood meals (in plaque-forming units per ml) are indicated. In each experiment a different triplet of mosquito families at the F2 (Experiment 2) or F3 generation (Experiments 1 and 3) were simultaneously challenged with the four isolates. (DOC) [file pgen.1003621.s013.doc]

**Table S2. Description of virus isolates.** The date of collection, serotype, number of passages in C6/36 cells, and measured infectious titers in the artificial infectious blood meals (in plaque-forming units per ml) are indicated. In each experiment a different triplet of mosquito families at the F_2_ (Experiment 2) or F_3_ generation (Experiments 1 and 3) were simultaneously challenged with the four isolates.

| Virus ID | Date isolated | Dengue  Serotype | Passage history | Blood meal titer (PFU/ml)  Experiment 1 | Blood meal titer (PFU/ml)  Experiment 2 | Blood meal titer (PFU/ml)  Experiment 3 |
| --- | --- | --- | --- | --- | --- | --- |
| DV3-10A | 3 March 2010 | DENV-3 | C6/36-3 | 1.0 x 10^5^ | 1.0 x 10^5^ | 1.5 x 10^5^ |
| DV3-14A | 30 March 2010 | DENV-3 | C6/36-3 | 1.5 x 10^5^ | 1.0 x 10^5^ | 3.0 x 10^5^ |
| DV1-26A | 15 June 2010 | DENV-1 | C6/36-3 | 1.0 x 10^6^ | 2.5 x 10^5^ | 1.5 x 10^6^ |
| DV1-30A | 6 July 2010 | DENV-1 | C6/36-3 | 7.5 x 10^5^ | 2.0 x 10^4^ | 4.5 x 10^5^ |
