# Supplementary material for: Genetic Mapping of Specific Interactions between Aedes aegypti Mosquitoes and Dengue Viruses
Source: PLoS Genet. 2013 Aug 1;9(8):e1003621. doi: 10.1371/journal.pgen.1003621 (PMC3731226; doi:10.1371/journal.pgen.1003621)
Supplement: Table S3 — Primers used for virus sequencing. Nucleotide positions and primer sequences are shown for each of the overlapping amplicons covering the viral genome. (DOC) [file pgen.1003621.s014.doc]

**Table S3. Primers used for virus sequencing.** Nucleotide positions and primer sequences are shown for each of the overlapping amplicons covering the viral genome.

| Serotype | Fragment | Forward primer 5’-3’ | Reverse primer 5’-3’ |
| --- | --- | --- | --- |
| DENV-3 | 1-1033 | agttgttagtctacgtg | ggtagtcacacaccccccgtg |
|  | 815-1770 | gcccttaggcacccagggtt | cccgcgaaaatgcttgtgc |
|  | 1398-2557 | cgcaaggagtcacggctgag | gcctgcaatggctgttgcc |
|  | 2155-3357 | aaggcgcatggccatcttg | gagcggcaacaccattcgtg |
|  | 3205-4062 | ggcaggaccctggcacttagg | gctgccactgtcattgggagc |
|  | 3969-5198 | cagccactctgattttggccg | caaccacccttgtcggtgcc |
|  | 5083-6083 | cataatggatcttcatcctgg | actcaccgtctatggcggctg |
|  | 5875-7216 | tgctgcgcaaaggagaggg | ttcacgggtggcttttgcttg |
|  | 6931-8213 | ctggacattgtacgccgtggc | tttcgtgcgtggagtttcgtg |
|  | 7879-9149 | aggcggtccaggacacgaag | tggctcctccgggtatcttgg |
|  | 8548-9559 | caaaccatgggatgtggtgcc | gcaatcatccccgctaatggc |
|  | 9356-10363 | aggggcagtggacaactggg | tttaacgtccttggacggggc |
|  | 9970-10707 | gtggatgactacagaaga | agaacctgttgattcaac |
| DENV-1 | 1-414 | agttgttagtctrygtgg | atgaggagcatggtcacagat |
|  | 41-1245 | cggaagcttgcttaacgtagttct | ccattaccccagcctctgtccac |
|  | 771-1570 | cctctgaaggcgcttggaa | ccattgtttgtggacgagcc |
|  | 1112-2344 | tgcattgaagccaaaatatcaaa | tgaatttaatcccaaccatgtc |
|  | 2189-3482 | gcatgggacttcggctctatagg | ctgaccctgcagagaccattga |
|  | 3391-4685 | aggagaagacggatgttggtatgg | ctcccctggtgacgtgccacatt |
|  | 4007-4806 | ttccctttatgcctgtccacg | tgcacttcttctcccgcgt |
|  | 4598-5870 | caaagaggactgttgggcagg | tcactggcatcggtccggcta |
|  | 5597-5998 | gtctggtttgttccaagca | ttttgcttctgtccaatgg |
|  | 5785-6956 | ggtaatagacccaaggcggtg | ctgcatagagagtccaggctgaag |
|  | 6658-7455 | gctatggatggccagtgtgg | ggagatccctcccaaagcg |
|  | 6850-8166 | cacaaagaaagacctggggattg | ctttgcatttgctccagagtttc |
|  | 7738-8538 | gtcgagaggaacggccaaac | ccattgaccatggatgaggc |
|  | 8058-9110 | tacgtgttctaaagatggtggaacc | tgtgcagtccttctccttccactc |
|  | 8858-9660 | cggttctgggaccttgtgc | gaaggttcccattgcggtatg |
|  | 8966-9965 | ggaaaggcaaaaggaagtcgtg | catggattgaccaggttgtg |
|  | 9787-10587 | agtatcacaaggcgccgga | ccaccagggtacagcttccc |
|  | 9858-10717 | agctgatgtacttccacaggaga | agcaccattccattttctgg |
|  | 10481-10735 | catggaagctgtacgcatgg | agaacctgttgattcaacagcacc |
